# Modeling gene expression using chromatin features in various cellular contexts

**DOI:** 10.1186/gb-2012-13-9-r53

**Published:** 2012-09-05

**Authors:** Xianjun Dong, Melissa C Greven, Anshul Kundaje, Sarah Djebali, James B Brown, Chao Cheng, Thomas R Gingeras, Mark Gerstein, Roderic Guigó, Ewan Birney, Zhiping Weng

**Affiliations:** 1 Program in Bioinformatics and Integrative Biology, Department of Biochemistry and Molecular Pharmacology, University of Massachusetts Medical School, Worcester, MA 01605, USA; 2 Department of Computer Science, Stanford University, 318 Campus Drive, Stanford, CA 94304, USA; 3 Centre for Genomic Regulation (CRG) and UPF, Dr. Aiguader, 88, 08003 Barcelona, Spain; 4 Department of Statistics, University of California, Berkeley, 367 Evans Hall, University of California, Berkeley, Berkeley, CA 94720, USA; 5 Computational Biology and Bioinformatics Program, Yale University, 266 Whitney Ave, New Haven, CT 06511, USA; 6 Cold Spring Harbor Laboratory, Genome Center, Woodbury, New York 11797, USA; 7 Vertebrate Genomics Group, European Bioinformatics Institute (EMBL-EBI), Wellcome Trust Genome Campus, Hinxton, Cambridgeshire, CB10 1SA, UK

## Abstract

**Background:**

Previous work has demonstrated that chromatin feature levels correlate with gene expression. The ENCODE project enables us to further explore this relationship using an unprecedented volume of data. Expression levels from more than 100,000 promoters were measured using a variety of high-throughput techniques applied to RNA extracted by different protocols from different cellular compartments of several human cell lines. ENCODE also generated the genome-wide mapping of eleven histone marks, one histone variant, and DNase I hypersensitivity sites in seven cell lines.

**Results:**

We built a novel quantitative model to study the relationship between chromatin features and expression levels. Our study not only confirms that the general relationships found in previous studies hold across various cell lines, but also makes new suggestions about the relationship between chromatin features and gene expression levels. We found that expression status and expression levels can be predicted by different groups of chromatin features, both with high accuracy. We also found that expression levels measured by CAGE are better predicted than by RNA-PET or RNA-Seq, and different categories of chromatin features are the most predictive of expression for different RNA measurement methods. Additionally, PolyA+ RNA is overall more predictable than PolyA- RNA among different cell compartments, and PolyA+ cytosolic RNA measured with RNA-Seq is more predictable than PolyA+ nuclear RNA, while the opposite is true for PolyA- RNA.

**Conclusions:**

Our study provides new insights into transcriptional regulation by analyzing chromatin features in different cellular contexts.

## Background

Gene expression refers to the process of producing a specific amount of gene product in a spatiotemporal manner. It is highly regulated in many steps, including transcriptional regulation, splicing, end modification, export, and degradation. Transcriptional regulation can occur on both genetic and epigenetic levels. Here, we define genetic regulation as a direct or indirect interaction between a gene and a transcription factor, and epigenetic regulation as altering DNA accessibility to transcription factors by chemical modification of chromatin. The basic unit of chromatin is structured like beads on a string, where the string is DNA and each bead is a DNA-protein complex called a nucleosome. Nucleosomes are an octameric complex of histone proteins composed of two copies of four core histones (H2A, H2B, H3 and H4) with roughly 147 bp of DNA wrapped around each octamer. Several post-translational modifications, such as methylation, acetylation, and phosphorylation, occur on the amino-terminal tails of histones. These modifications can change the structure and function of chromatin by recruiting other enzyme complexes [1]. It has been proposed that these histone modifications can occur combinatorially to form a 'histone code' that is read by other proteins to give rise to various downstream events such as transcription [2,3].

Histone modifications have been shown to be involved in both activation and repression of transcription. Early studies on individual modifications reported their function in transcription regulation. For example, H3K4me1 [4] and H3K4me3 [5] are associated with transcriptional activation, while H3K9me3 and H3K27me3 are associated with transcriptional repression [6]. Wang *et al*. [7] systematically analyzed 39 histone modifications in human CD4^+ ^T cells and found that histone acetylation positively correlates with gene expression, consistent with its role in transcriptional activation. By clustering histone modification patterns into classes, they also showed that the class with the lowest expression contains H3K27me3 but no acetylation, the class with intermediate expression contains H3K36me3, a backbone of 17 modifications, or the backbone plus H4K16ac, and the class with the highest expression contains H2BK5me1, H4K16ac, H4K20me1, and H3K79me1/2/3 in addition to the backbone. The correlation between histone modifications and expression is also found in yeast [8] and *Arabidopsis thaliana *[9]. Using the same datasets as the Wang *et al*. study [7], Karlić *et al*. [10] recently derived quantitative models to predict gene expression using histone modifications and showed that they are well-correlated. Cheng *et al*. [11] derived a support vector machine model from modENCODE worm data and applied it to human K562 cells and mouse embryonic stem cells with good performance (Pearson's correlation coefficient (PCC) *r = *0.73 and 0.74, respectively). Both studies successfully quantified the relationship between histone modifications and gene expression. However, due to the limited human datasets used in these studies (for example, only one cell line and/or no information regarding RNA type), it is still largely unknown if this relationship remains true in other cellular contexts.

Here, we further study this relationship taking advantage of the wealth of datasets from the ENCODE project [12,13]. We analyzed genome-wide localization for eleven histone modifications, one histone variant, and DNase I hypersensitivity in seven human cell lines (see Materials and methods). For each cell line, ENCODE members extracted RNA (for example, PolyA+, PolyA-) using different protocols from different cellular compartments (for example, whole cell, nuclear, cytosolic), and measured their levels using various techniques (cap analysis of gene expression (CAGE), RNA paired-end tag (RNA-PET) sequencing, and RNA-Seq), thus providing us an excellent platform for studying the relationship between chromatin features and gene expression across different cellular contexts. We set out to answer the following questions. First, can we reproduce the quantitative relationship between gene expression levels and histone modifications? Second, does the relationship hold across different human cell lines and between different groups of genes? Third, if so, do the most predictive chromatin features differ depending on the expression quantification technique used? And fourth and more interestingly, how well can the chromatin features predict expression levels of RNA from different cell compartments and/or RNA extracted by different methods (such as PolyA+ versus PolyA-)? To address these questions, we derived a novel two-step quantitative model to correlate measured gene expression levels with histone modification levels. Our model not only confirms the general relationship between histone modifications and transcription output shown in previous studies [10,11], but also shows that correlation strength and the most predictive chromatin features vary when different techniques were used for quantifying expression. For example, transcriptomes quantified by CAGE are better predicted by promoter marks such as H3K4me3, whereas structural marks like H3K79me2 and H3K36me3 are better predictors for transcriptomes measured with RNA-Seq. Consistent with previous studies, low CpG genes are shown to be less predictable than high CpG genes, and these two groups of genes differ in their sets of predictive chromatin features. This study also shows previously unknown results, such as that PolyA+ RNA is more predictable than PolyA- RNA, and for RNA-Seq based measurement, cytosolic RNA is more predictable than nuclear RNA for PolyA+, while the reverse is true for PolyA-. In summary, using the wealth of data from the ENCODE project, our analysis not only confirms the quantitative relationship between chromatin features and gene expression via a powerful model, but further provides a more comprehensive and accurate view on this relationship by comparing the model's performance in different cellular contexts.

## Results

### Development of a new quantitative model to correlate chromatin features with transcription levels

To further understand the relationship between chromatin features and expression levels under various conditions, we took advantage of the massive high-throughput sequencing data from the ENCODE Consortium [12], which includes genomic localization data for eleven histone modifications and one histone variant in seven human cell lines [14], and expression quantification data for various cell compartments and RNA extractions (for example, PolyA+, PolyA-) in each corresponding cell line (see Materials and methods). Moreover, gene expression levels were quantified in two forms: RNA-Seq [15] was used to quantify transcript (Tx)-based expression levels; and CAGE [16,17] and 5' tags of RNA-PET [18] were used to capture transcription start site (TSS)-based expression levels [19]. Thus, CAGE best captures the transcriptional initiation of genes while RNA-Seq profiles transcription elongation. For comparison, we also derived TSS-based expression levels by summing the RNA-Seq quantification for transcripts that share the same TSS.

Previous studies used a mean signal of the TSS-flanking region ([-2k, +2k] around the TSS) [10,20] to estimate the level of histone modifications for a gene. However, this strategy could result in bias since modification marks have different density distributions along the gene [11]. For instance, H3K4me3 and H3K36me3 peak at 5' and 3' ends, respectively [21]. To better estimate the representative signal for each chromatin feature, we divided specific genetic regions into bins following the approach by Cheng *et al*. [11] and searched for the bin(s) showing the best correlation between the chromatin feature signal and the expression level, namely 'bestbin'. The bestbin was determined using one-third of all genes (D1) and applied to the remaining two-thirds of genes (D2) for further analysis (see Materials and methods).

**Figure 1:**
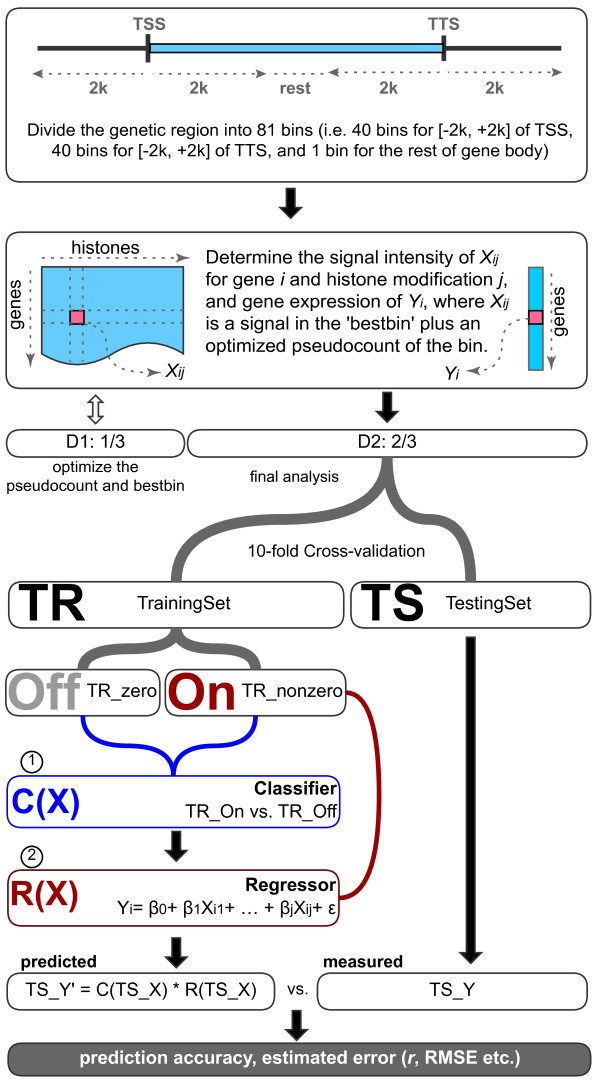
**Modeling pipeline**. Genes longer than 4,100 bp were extended and divided into 81 bins. The chromatin feature density in each bin is logarithm-transformed and then used to determine the best bin (the bin that has the strongest correlation with the expression values). To avoid log2(0), a pseudocount is added to each bin, which is then optimized using one-third of genes in each dataset (D1) and then applied to the other two-thirds of genes in the datasets (D2) for the rest of the analysis. D2 was divided into training set (TR) and testing set (TS) in a ten-fold cross-validation manner. A two-step model was built using the training set. First, a classification model C(X) was learned to discriminate the 'on' and 'off' genes, followed by a regression model R(X) for predicting the expression levels of the 'on' genes. Finally, the correlation between the predicted expression values for testing set, C(TS_X)*R(TS_X), and the measured expression values of testing set (TS_Y) was used to measure the overall performance of the model. TSS, transcription start site; TTS, transcription termination site; RMSE, root-mean-square error.

We used a two-step model to determine the correlation between chromatin features and expression levels (Figure 1; see Materials and methods for more details). Briefly, we first transformed the normalized tag counts X*_ij _*for chromatin feature *j *at gene *i *to a logarithmic scale log2(X*_ij_*). To avoid the issue of log2(0), a pseudocount a*_j _*optimized using D1 was added to the same modification in D2. The result of 'bestbin' selection and the corresponding pseudocount for each chromatin feature is shown in Table S1 in Additional file 1. We then built models to predict logarithm-scaled expression values log2(Y*_i_*) using the log2(X*_ij _*+ a*_j_*) of each chromatin feature on the remaining dataset of D2. We performed ten-fold cross-validation on D2 to verify that the correlation was not specific to a subset of data. Considering the structure of the data, we first trained a random forests classifier C(X) to distinguish the genes with expression level of 0 ('off') from the non-zero ('on') genes and a regressor R(X) on the non-zero genes in the training set, and then applied C(X)*R(X) to the test set. In addition to the linear regression model, we also applied non-linear models such as multivariate adaptive regression splines (MARS) and random forests for the regressor. The Pearson's correlation coefficient (*r*) and normalized root-mean-square error (RMSE) between the overall measured and predicted expression values were then calculated to assess correlation. Our model shows excellent correlation between chromatin features and expression levels for both TSS-based and Tx-based data.

**Figure 2:**
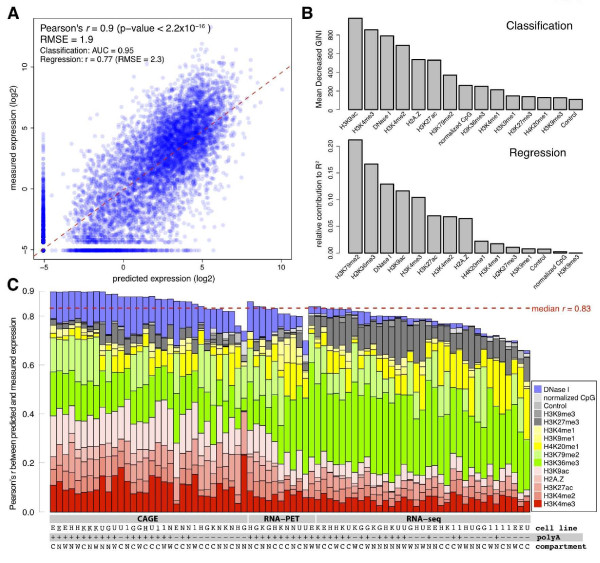
**Quantitative relationship between chromatin feature and expression**. **(a) **Scatter plot of predicted expression values using the two-step prediction model (random forests classification model and linear regression model) versus the measured PolyA+ cytosolic RNA from K562 cells measured by CAGE. Each blue dot represents one gene. The red dashed line indicates the linear fit between measured and predicted expression values, which are highly correlated (PCC *r *= 0.9, *P*-value <2.2 × 10^-16^), indicating a quantitative relationship between chromatin features and expression levels. The accuracy for the overall model is indicated by RMSE (root-mean-square error), which is 1.9. Accuracy for the classification model is indicated by AUC (area under the ROC curve), which is 0.95. The accuracy for the regression model is *r *= 0.77 (RMSE = 2.3). **(b) **The relative importance of chromatin features in the two-step model. The most important features for the classifier (upper panel) include H3K9ac, H3K4me3, and DNase I hypersensitivity, while the most important features for the regressor (bottom panel) include H3K79me2, H3K36me3, and DNase I hypersensitivity. **(c) **Summary of overall prediction accuracy on 78 expression experiments on whole cell, cytosolic or nuclear RNA from seven cell lines. The bars are sorted by correlation coefficient in decreasing order for each high throughput technique (CAGE, RNA-PET and RNA-Seq). Each bar is composed of several colors, corresponding to the relative contribution of each feature in the regression model. The red dashed line represents median PCC *r *= 0.83. Code for cell lines: K, K562; G, GM12878; 1, H1-hESC; H, HepG2; E, HeLa-S3; N, NHEK; U, HUVEC. Code for RNA extraction: +, PolyA+; -, PolyA-. Code for cell compartment: W, whole cell; C, cytosol; N, nucleus.

Figure 2a shows one example where CAGE performed on long cytosolic PolyA+ RNA from K562 cells shows an overall high prediction accuracy with PCC *r = *0.9 and a *P*-value <2.2 × 10^-16^. Note that many genes (approximately 6,000 in Figure 2a) have a zero expression level and are correctly classified as unexpressed. These genes appear as a single dot at the lower left corner of the graph, without which the PCC would be lower (see below). We also measured the accuracy and importance of chromatin features for classification and regression. We correctly classified 90.44% of genes into 'on' and 'off' categories (area under the receiver operating characteristic (ROC) curve (AUC) = 0.95; Figure S1A in Additional file 2), and achieved PCC *r = *0.77 and RMSE = 2.30 for regressing the 'on' genes. Diagnostic analysis of residuals also shows that the normality assumption is satisfied (Figure S1B,C in Additional file 2).

In addition to the logarithm transformation, we also converted the expression values to ranked 'normal scores' using the rankit transformation, which obviates the need of a pseudocount (see Materials and methods). We still saw significant correlation between predicted and measured normal scores (Figure S1D in Additional file 2; *r = *0.86, RMSE = 0.71). In addition to the linear regression model, we used two other multivariate regression models (MARS and random forests), which automatically model non-linearity. These three methods show similar prediction accuracies (Figure S2 in Additional file 2) and we thus chose the simplest linear model for the rest of our analysis. We also used a random sampling method to ensure that the prediction accuracy is stable and independent of sample size (Figure S3 in Additional file 2).

We determined the relative importance of each feature for predicting expression datasets (see Materials and methods). We observed that histone modifications like H3K9ac and H3K4me3 are more important in identifying genes that are 'on' or 'off,' while histone modifications like H3K79me2 and H3K36me3 are more important for regression of expressed genes (Figure 2b). DNase I hypersensitivity is the third most important feature for both classification and regression. We also observed that the normalized CpG score is more important for gene 'on' or 'off' status classification than for regression of the expression levels of 'on' genes. This is consistent with the observation that the percentage of high CpG promoter genes increases along with increasing average expression levels of the genes (Figure S4B in Additional file 2).

To verify that there are no inherent structures in the data that can lead to an 'easy' prediction, we performed three randomization tests for each prediction. First, we randomly shuffled expression values (Y) of genes without shuffling chromatin and sequence features (X), which gives a baseline performance based on random assignments of promoters to genes, which, as expected, yielded a very low PCC (*r = *0.01) and a high RMSE (5.51). In the second randomization test, we shuffled each chromatin feature independently (without changing the labels for the chromatin features). This also led to low accuracy (*r = *-0.01, RMSE = 6.27). In the third test we swapped the × labels before applying the models to the testing set to check the importance of having an accurate coefficient for each chromatin feature. Again, this led to lower accuracy (*r = *0.57, RMSE = 3.30). The residual correlation is likely due to correlations between some chromatin features.

We summarized the correlation coefficients between predicted and measured expressions for all 78 RNA expression experiments from the seven cell lines in our analysis (Figure 2c). It shows that most experiments show a strong correlation (median *r *= 0.83) between predicted and measured expression levels by both TSS-based CAGE and RNA-PET and Tx-based RNA-Seq techniques. Table S2 in Additional file 1 contains a detailed display for each experiment, including the correlation coefficient, *P*-value for the correlation, the individual correlation, and relative importance of each chromatin feature. In the remaining sections, we analyze the performance of our models according to techniques for measuring expression, cell line, types of chromatin features, types of TSS, and cellular compartment.

### Comparison of different techniques for measuring expression

**Figure 3:**
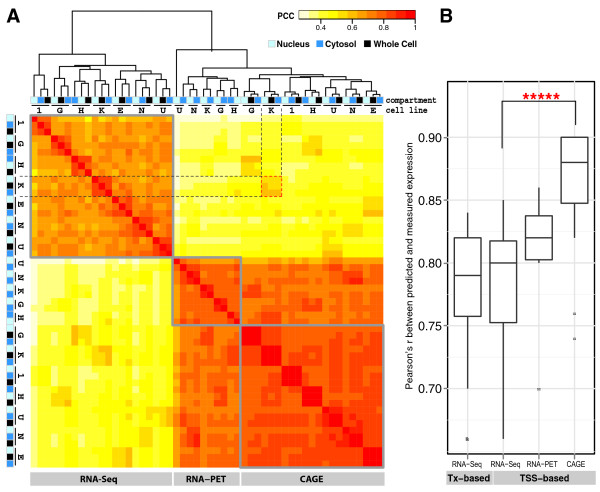
**Comparison of expression quantification methods**. **(a) **Heatmap of correlations between PolyA+ experiments from various cell lines and cell compartments. Experiments from the same expression quantification methods tend to cluster together, and CAGE and RNA-PET are closer to each other than they are to RNA-Seq. The clustering tree also shows that experiments on different cell compartments in the same cell line tend to group together and RNA expression from the cytosol (blue) and whole cell (black) tend to group together rather than with that of the nucleus (light blue). Code for cell lines: K, K562; G, GM12878; 1, H1-hESC; H, HepG2; E, HeLa-S3; N, NHEK; U, HUVEC. **(b) **Boxplot of correlation coefficients for all expression prediction in CAGE, RNA-PET, and RNA-Seq categories. Paired Wilcoxon test shows that CAGE-based expression data are significantly better predicted than RNA-Seq-based expression data (*P*-value = 3 × 10^-5^).

Due to high correlation between replicates (Figure S5 in Additional file 2), we merged multiple replicates from the same sample into one dataset. After merging, there were a total of 39, 14, and 45 expression datasets in the CAGE, RNA-PET, and RNA-Seq categories, respectively (Table S3 in Additional file 1). Out of the 98 total experiments, 78 were done for PolyA+ or PolyA- RNAs from whole cell, cytosol or nucleus. We first compared the expression levels measured by these three different techniques. By clustering long PolyA+ RNA measurements from seven cell lines with measurements from three cellular compartments for each cell line, we see that experiments using the same technique tend to group together, and that RNA-Seq is an out-group of CAGE and RNA-PET (Figure 3a). Nonetheless, RNA-Seq expression is positively correlated with CAGE and RNA-PET expression for RNA extracted from the same cell line (for example, *r = *0.57 between CAGE and RNA-Seq measurements for cytosolic PolyA+ RNA from K562 cells; see the 3 × 3 red dashed box in Figure 3a). The correlation increases when considering only single-transcript genes (*r = *0.69 for the same example; Figure S6 in Additional file 1). An assessment of RNA from different cellular compartments in the same cell line shows that whole cell extracted RNA is more similar to cytosolic RNA than nuclear RNA (Figure 3a). This may be due to the presence of a poly(A) tail, which aids in exporting mRNA from the nucleus, and offers protection from cytoplasmic degradation.

We applied our models to each dataset to determine the prediction accuracy, measured as the correlation between predicted and measured expression levels. To compare the prediction accuracy of these different expression datasets, we grouped all PolyA+ experiments from the same high throughput technique and Figure 3b shows the distributions of the correlation coefficients. We see that expression measured by each of the three techniques is well-predicted by the model (median *r *ranges from 0.79 to 0.88), although, on average, predictions for expression from CAGE are better than for RNA-PET or RNA-Seq (Figure 3b). We also observed that both TSS-based and Tx-based RNA-Seq quantifications have comparable performance (median *r = *0.80 and 0.79, respectively) for all genes (Figure 3b) as well as for single-transcript genes only (data not shown), indicating that the lower predictivity for RNA-Seq is not due to multiple transcripts that share the same TSS. For subsequent analysis, we used RNA-Seq data only for Tx-based expression.

### Chromatin features are predictive of gene expression across different ENCODE human cell lines

**Figure 4:**
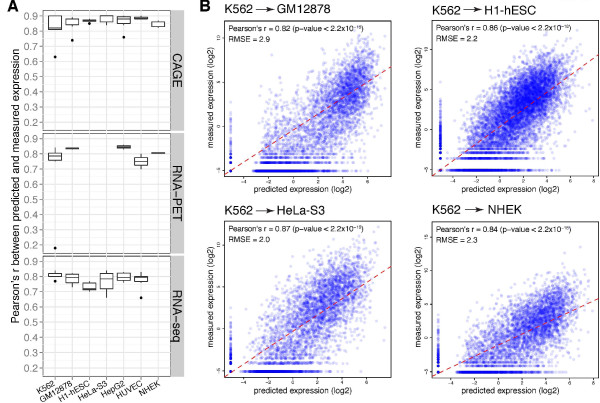
**Comparison of prediction accuracy across different cell lines**. **(a) **Boxplot of correlation coefficients for seven cell lines (K562, GM12878, H1-hESC, HeLa-S3, HepG2, HUVEC and NHEK) with different types of expression quantification (CAGE, RNA-PET, and RNA-Seq). It shows that the high quantitative relationship between chromatin features and expression exist in various cell lines and using different expression quantification methods. Paired Wilcoxon tests between H1-hESC and other cell lines show that H1-hESC has significantly lower prediction accuracy (*P*-value = 0.02, 0.02, 0.07, 0.02, and 0.05 for K562, GM12878, HeLa-S3, HepG2 and HUVEC, respectively). **(b) **Application of the model learned from K562 to other cell lines (GM12878, H1-hESC, HeLa-S3 and NHEK) indicates that the model performs well across cell lines (*r *= 0.82, 0.86, 0.87 and 0.84, respectively). This indicates that the quantitative relationship between chromatin features and gene expression is not cell line-specific, but rather a general feature.

We then compared different cell lines to see whether gene expression is better predicted by chromatin features in some cell lines over others. Figure 4a shows PCCs for seven cell lines, both for TSS-based CAGE data and Tx-based RNA-Seq data, with an average *r *of 0.8 (with a relatively lower correlation for RNA-Seq data from the H1-hESC cell line; see discussion below). This shows that our models are effective at predicting gene expression by chromatin feature signals among various cell lines.

To further explore whether the models are generalizable across different cell lines, we applied the model trained in one cell line to other cell lines, using the values of chromatin features in those cell lines as inputs to the models to determine if the prediction accuracy dramatically changed. Figure 4b shows an example of this cross-cell line prediction, wherein we learned a prediction model from CAGE-measured PolyA+ cytosolic RNA from K562 cells and applied it to CAGE-measured PolyA+ cytosolic RNA from four other cell lines. The prediction accuracy remains high, with *r = *0.82, 0.86, 0.87, and 0.84 for GM12878, H1-hESC, HeLa-S3, and NHEK cell lines, respectively. These results indicate that our models accurately captured the relationships among the various chromatin features and are broadly applicable to predicting expression in all cell lines.

Even though the models work well for different cell lines, we observed that H1-hESC cells have relatively weaker correlations than the other six cell lines for predicting RNA-Seq-based experiments, unlike in CAGE-based experiments, where all seven cell lines have equally high correlations (Figure 4a). This may be due to a difference in transcriptome features between undifferentiated stem cells and committed cells. Transcriptional pausing (that is, initiation but no elongation) is an obligate transition state between definitive activation and silencing, as the cell changes from an undifferentiated to a committed state [22]. A study comparing mouse embryonic stem cells with mouse embryonic fibroblasts also showed that, during differentiation, many genes leave the paused state and enter the elongation state [23]. While our model cannot directly compare H1-hESC with other cell lines based on differentiation, our results are in line with the observation that many genes in H1-hESC are transcriptionally paused, and thus more precisely captured by CAGE, while eluding full capture by RNA-Seq.

### Transcription initiation and elongation are reflected by different sets of chromatin features

In addition to determining the chromatin features that contribute the most to individual expression datasets (as shown in Figure 2b), we also wanted to determine if different types of chromatin features contribute the most in predicting CAGE-measured RNA, polyadenylated RNA, and RNA from a specific cellular compartment, and so on. To do so, rather than analyzing all possible combinations of chromatin features, we simply grouped the eleven histone marks and one histone variant into four categories based on their known functions in gene regulation, namely, H3K4me2, H3K4me3, H2A.Z, H3K9ac and H3K27ac as promoter marks [5,24], H3K36me3 and H3K79me2 as structural marks [25,26], H3K27me3 and H3K9me3 as repressive marks [6], and H3K4me1, H4K20me1 and H3K9me1 as distal/other marks [4,6]. These groupings allow us to determine the prediction accuracy based upon each category, as well as combinations of different categories (such as promoter and structural marks together).

**Figure 5:**
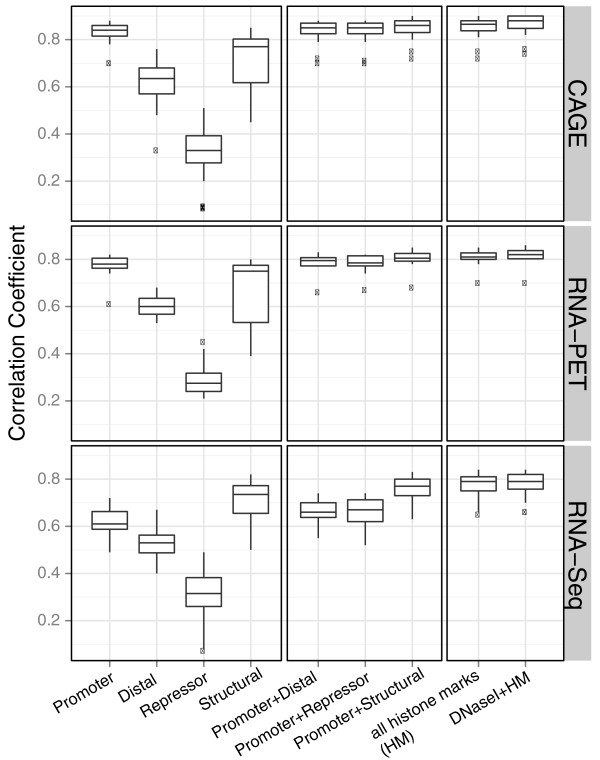
**Comparison of groups of chromatin features**. Twelve chromatin features are grouped into four categories according to their known function in gene regulation: promoter marks (H3K4me2, H3K4me3, H2A.Z, H3K9ac, and H3K27ac), structural marks (H3K36me3 and H3K79me2), repressor marks (H3K27me3 and H3K9me3), and distal/other marks (H3K4me1, H4K20me1, and H3K9me1). Correlation coefficients are shown for individual categories, a combination of promoter with three other categories, all histone marks (HM), and HM together with DNase I hypersensitivity are shown in the boxplot for CAGE (TSS-based), RNA-PET (TSS-based), and RNA-Seq (Tx-based) expression data. It indicates that for TSS-based data, promoter marks are the most predictive among the four categories, while for Tx-based expression, structural marks are the most predictive.

By comparing the prediction accuracy using marks from each category or a combination of two categories (Figure 5), we show that for CAGE TSS-based gene expression, promoter marks are the most predictive, while for RNA-Seq Tx-based expression data, structural marks are better predictors. For CAGE-measured PolyA+ cytosolic RNA, promoter marks as a group have high correlation coefficients (median *r = *0.86). Promoter marks combined with another category of chromatin features give equally high prediction accuracy. However, non-promoter mark categories have lower prediction accuracy (for example, median *r = *0.84 for structural marks only; median *r = *0.35 for repressive marks only). On the other hand, structural marks like H3K79me2 and H3K36me3 are more predictive for RNA-Seq expression data. This was expected, since CAGE mainly profiles transcription initiation events and RNA-Seq captures transcription elongation. Thus, our results further confirmed that transcription initiation and elongation are characterized by different chromatin marks. We noticed that DNase I hypersensitivity, a general indicator for open chromatin, has a significantly lower correlation coefficient (*r = *0.83, paired Wilcoxon test *P*-value = 4 × 10^-15^) than that of promoter marks. This is also observed in other experiments (Figure S7 in Additional file 2), and may indicate that open chromatin is a general prerequisite for regulating gene expression, but that histone modifications are involved in fine-tuning expression levels.

### Genes with high CpG content promoters are more predictable than those with LCP promoters

**Figure 6:**
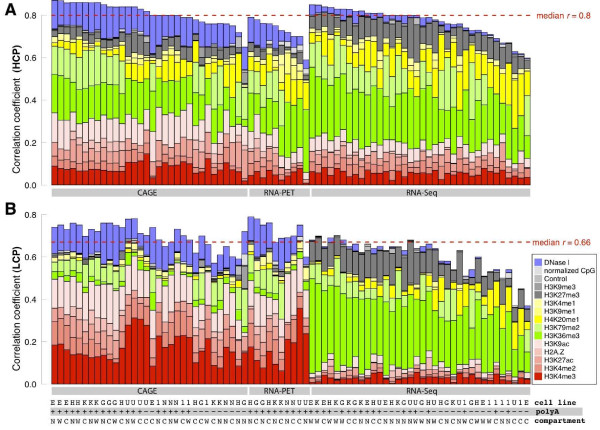
**Comparison of the prediction accuracy of high- and low-CpG content promoter gene categories**. **(a) **Summary of prediction accuracy for all high-CpG content promoter (HCP) genes in 78 RNA expression experiments on whole cell, cytosolic or nuclear RNA, showing that the median correlation for all experiments is *r *= 0.8. Each bar is divided into different colors corresponding to the relative contribution of variables in the regression model. **(b) **Same as in (a), but for low-CpG content promoter (LCP) genes, showing that the median correlation coefficient for all experiments is *r *= 0.66. This indicates that HCP genes are better predicted than LCP genes. Comparison of the relative contribution of various chromatin features in each experiment indicates that the promoter marks (red and light red) show more importance in predicting LCP genes using TSS-based data (for example, CAGE and RNA-PET), while structural marks (green show most importance in predicting LCP genes for transcript-based data. Code for cell lines: K, K562; G, GM12878; 1, H1-hESC; H, HepG2; E, HeLa-S3; N, NHEK; U, HUVEC. Code for RNA extraction: +, PolyA+; -, PolyA-. Code for cell compartment: W, whole cell; C, cytosol; N, nucleus.

Previous studies have shown that CpG-rich promoters are associated with ubiquitously expressed genes while CpG-poor (and often TATA-containing) promoters are associated with cell type-specific genes [27-29] and have different patterns of histone modifications [29]. We expected that the predictive power of chromatin features based on ENCODE data would differ between the genes driven by high CpG content promoters (HCPs) or low CpG content promoters (LCPs). To test this, we divided genes into two groups based on their normalized CpG score in the promoter region (see Materials and methods), and applied our models on both groups. The results show that the models have higher prediction power on HCP genes than on LCP genes for most of the experiments (median *r = *0.8 for HCP versus 0.66 for LCP, *P*-value = 2.19 × 10^-14^; Figure 6), independent of high throughput technique or chromatin feature category (Figure S4A in Additional file 2).

We also examined whether different sets of chromatin features are necessary for predicting the expression of HCP and LCP genes. The most important chromatin features for HCP genes are similar to those for all genes (compare Figures 6a and 2c), consistent with the finding from previous work [10]. We noticed that H3K79me2 and H3K36me3 are the top two predictors for HCP genes and all genes. Promoter marks (the red group in Figures 2c and 6) are more important for CAGE and RNA-PET measured transcriptomes whereas structural marks (the green group) are important for RNA-Seq measured transcriptomes. Strikingly, this difference becomes more pronounced in LCP genes (Figure 6b), where H3K4me3 and H3K9ac are the top two predictors for CAGE and RNA-PET measured transcriptomes, and H3K36me3 is far more important for predicting the RNA-Seq measured transcriptomes. Again, the Tx-based RNA-Seq dataset allows us to measure the chromatin feature signal along the whole gene body until the 3' end, where structural marks like H3K36me3 were shown to have strong signals. This explains why H3K36me3 is a more important mark for RNA-Seq expression than for CAGE or RNA-PET. However, it is unclear why the difference is so much greater in LCP genes. We venture to suggest that the regulation of the transcription initiation and elongation are uncoupled for LCP genes, and the chromatin features that are most predictive for initiation are thus poor predictors of elongation, and vice versa.

We compared our most predictive chromatin features to the HCP and LCP expression predictions by Karlić *et al*. [10]. While their datasets and methods to measure the relative importance of chromatin features differed from ours, the lists for the top effectors partially overlap. For example, H3K4me3 is important for LCPs and H4K20me1 shows greater importance for HCPs than LCPs.

Since LCP genes typically have low expression levels, we compared the predictability of highly and lowly expressed genes to establish if there are differences in the most predictive chromatin features. Genes were divided into ten bins according to their expression levels measured by CAGE, and we calculated the prediction accuracy in a cumulative way. Results show that the percentage of LCP genes anti-correlate with expression levels, confirming that more of the LCP genes fall into the category of lowly expressed genes. The relative importance of various marks in different subsets of genes also indicates that structural marks like H3K79me2 and H3K36me3 are better at predicting highly expressed genes while promoter marks become more predictive when lowly expressed genes are added (Figure S4B in Additional file 2). This is consistent with our previous observations that structural marks are more important in predicting HCP genes while promoter marks are more important in predicting LCP genes using CAGE quantification (Figure 6).

### Comparison of different RNA types in different cell compartments

Current high-throughput sequencing methods largely rely on the enrichment of transcripts with a Poly(A) tail, which precludes analysis of the expression and regulation of PolyA- transcripts. On the other hand, PolyA- RNAs have important biological functions. Katinakis *et al*. [30] suggested that some transcripts can be 'bimorphic' (that is, existing in both PolyA+ and PolyA- forms), and that PolyA+ transcripts can be processed to reduce or totally remove the Poly(A) tail under certain conditions. A recent study confirmed the existence of bimorphic transcripts in two human cell lines, and showed dynamic expression of a subset of PolyA- histone mRNA during differentiation [31]. While the regulation of PolyA- RNAs is far from fully understood, it is possible that PolyA+ and PolyA- RNAs are regulated by different mechanisms.

We first compared expression levels of PolyA+ RNAs and PolyA- RNAs among different cell compartments, such as whole cell, cytosolic, and nuclear. As described above, Figure 3a shows the clustering of all long PolyA+ RNA expression levels for all genes measured by different techniques, and whole cell and cytosolic RNA cluster together while nuclear RNA is an out-group. Clustering all PolyA+ and PolyA- RNA from RNA-Seq experiments (Figure S8 in Additional file 2) shows that PolyA- RNA is largely different from PolyA+ RNA. Interestingly, unlike the high similarity in expression levels between PolyA+ RNA from different compartments within the same cell line, expression levels from PolyA- cytosolic RNA are more similar across different cell lines than compared with PolyA- RNA from nuclear or whole cell extracts in the same cell line. On the other hand, whole cell and nuclear PolyA- RNA from the same cell line cluster together, consistent with the knowledge that most PolyA- RNAs reside in the nucleus.

**Figure 7:**
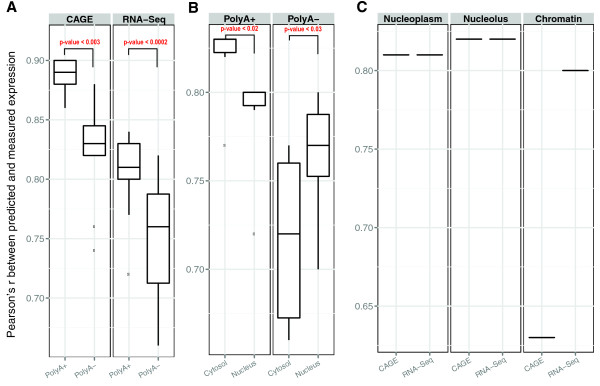
**Comparison of prediction accuracy among different RNA extractions and different cell compartments**. **(a) **Prediction accuracy of PolyA+ and PolyA- RNA for all genes measured with the CAGE and RNA-Seq techniques. This shows that PolyA+ RNA are better predicted than PolyA- RNA (*P*-value of paired Wilcoxon test between PolyA+ and PolyA-). **(b) **Prediction accuracy of PolyA+ and PolyA- RNA from different cell compartments for all genes measured with the RNA-Seq technique (*P*-value of paired Wilcoxon test between cytosol and nuclues). **(c) **Prediction accuracy of total RNA in different nuclear sub-compartments, measured by CAGE or RNA-Seq.

We then assessed how well histone modifications can predict PolyA+ and PolyA- RNA levels. PolyA+ RNA is significantly better predicted than PolyA- RNA, regardless of the technique with which RNA levels are measured and the location from which the RNA molecules are extracted (Figure 7a,b), indicating that the PolyA- fraction might be regulated by different mechanisms from the PolyA+ fraction. We also compared the performance for RNAs extracted from different compartments. The analysis based on RNA-Seq datasets showed that for polyadenylated RNAs (left panel of Figure 7b), cytosolic RNA is significantly better predicted than nuclear RNA (paired Wilcoxon test *P*-value = 0.01) and the reverse is true for non-polyadenylated RNA (*P*-value = 0.03). We noticed that the better predicted RNA populations (PolyA- nuclear RNA and PolyA+ cytosolic RNA) comprise the majority of their respective mRNA populations. Chromatin features were less predictive of the other two minority groups (PolyA+ nuclear RNA and PolyA- cytosolic RNA), possibly because degradation plays an important role in their abundances, and degradation is not accounted for in our model.

We further looked into the performance of nuclear sub-compartments (chromatin, nucleoplasm, and nucleolus). The nucleus is the largest cellular organelle in animals, and is composed of a nuclear envelope, chromatin, a nucleolus, and nucleoplasm (similar to the cytoplasm found outside of the nuclear envelope). Using the total RNA extracted from K562 cells, we showed that the RNAs from the three sub-compartments have comparable prediction accuracy between CAGE and RNA-Seq (Figure 7c), with the exception of chromatin-associated RNAs. We noticed that the chromatin RNAs measured by RNA-Seq are much better predicted than those measured by CAGE (*r = *0.8 versus 0.63), which might indicate that chromatin-associated RNA is transcribed, but uncapped.

## Discussion

In this study, we have derived a novel two-step model to study the relationships between chromatin features and gene expression. With this model, we have shown strong correlation (for example, *r = *0.9) between gene expression and chromatin features in various human cell lines, confirming the conclusions from previous studies with better performance. We also took advantage of the wide range of datasets from the ENCODE project and compared the accuracy of predicting RNA measured by different sequencing techniques (that is, CAGE, RNA-PET, and RNA-Seq), and from different cell lines (for example, embryonic stem cells, normal tissue cells, and tumor cells) and different cell compartments. We showed that different groups of chromatin features reflect gene 'on'/'off' status versus gene transcription levels. Also, we revealed different groups of chromatin features predict CAGE- versus RNA-Seq-based expression, suggesting transcription initiation and transcription elongation are represented by different sets of chromatin features. Comparisons among various cellular sub-compartments suggests that the non-polyadenylated RNAs might be regulated by different mechanisms from polyadenylated RNAs, and that chromatin-associated RNAs are likely transcribed, but uncapped.

Although previous studies have already identified the correlation between chromatin features and gene expression levels, our study makes additional contributions in three ways. First, our analysis benefits from the wealth of data produced by the ENCODE project, allowing us to use the widest range of data thus far to study this problem. The ENCODE Consortium quantified RNA species in whole cells and sub-cellular compartments, mapped histone modifications by ChIP-Seq, and measured chromatin and DNA accessibility in various cell lines. Unlike the limitations of other studies (for example, only one cell line, no RNA type), for the first time we have linked gene expression with its effectors in great detail and in well-matched conditions.

Second, we built a novel two-step model to quantify the relationship between chromatin features and expression. Several early studies [7,32-38] either simply described this relationship or quantified chromatin features and/or expression. Recent studies [10,11,39] have assessed the relationship using more sophisticated quantitative models. Here, our model expands upon this previous work by using both classification and regression, giving an even further in-depth analysis of the relationship. Given the observation that nearly 40% of all TSSs are not expressed in each of the investigated datasets (data not shown), applying regression directly on a dataset with many zeros could bias the result. Compared with a regression model alone, the two-step model shows an improvement in performance (for example, *r *= 0.895 versus 0.871 for the dataset in Figure 2a; Table 1). More importantly, chromatin features involved in turning gene expression 'on' and 'off' may differ from those that control the level of expression. This is why we chose a two-step model - first classifying the 'on' and 'off' genes by the available features, then performing regression on the expressed genes only - so each predicted expression is based on the product of the output of these two models. Additionally, instead of using a fixed bin for different chromatin features, we used the 'bestbin' strategy to capture maximal effects from different chromatin features. We have compared the performance of the 'bestbin' strategy with that of several other bin-selection methods. Table 1 shows that the 'bestbin' approach improves the performance by 2 to 13% compared to fixed-bin or no binning, and that 'bestbin' has the best performance overall. Moreover, most chromatin marks show very stable 'bestbin', such as H3K36me3, DNase, H3K27me3, H4K20me1, and H3K9me1 (Figure S9 in Additional file 2). Finally, using an optimal pseudocount led to a consistent improvement in performance compared with using a small fixed pseudocount (Figure S10 in Additional file 2), without changing the primary conclusions.

**Table 1:** Performance of different modeling and bin selection strategies

	Allbins	TSSbin	bins.0.2	best5bins	bestbin
Simple model	0.772 (2.77)	0.836 (2.40)	0.770 (2.78)	0.867 (2.16)	0.871 (2.14)
Two-step model	0.839 (2.37)	0.877 (2.10)	0.841 (2.36)	0.889 (1.99)	0.895 (1.94)

Simple models only perform regression, whereas our two-step model performs classification before regression. The columns are different bin-selection strategies, where 'allbins' uses the mean density of all bins, 'TSSbin' uses the two bins flanking the TSS, 'bins.0.2' uses the bins with individual correlation coefficient (*r*) greater than a threshold (0.2 in this case), 'best5bins' uses the top five bins with the greatest *r*, and 'bestbin' uses the bin(s) with the greatest *r*. The values are PCCs (*r*) between predicted and measured expression levels of PolyA+ cytosolic RNA from K562 cells measured by CAGE, and the values in brackets are RMSE for the predictions.

Third, our model performs well in predicting gene expression using chromatin features. Using a linear regression model to correlate histone modifications at promoters and expression in human CD4^+ ^T cells, Karlić *et al*. [10] calculated a correlation coefficient of *r = *0.77 for microarray data, and 0.81 for RNA-Seq data. Cheng *et al*. [11] showed that a support vector machine regression model learned from modENCODE worm data has *r = *0.73 in human K562 cells, and *r = *0.74 in mouse embryonic stem cells. Our model expands upon these well-performing models, with a number of datasets having *r *> 0.9, and 55 (out of 78) datasets having *r *≥ 0.8.

While our model shows high correlation between chromatin features and gene expression levels, it cannot be used to imply the causal effect of chromatin features on gene expression. Henikoff and Shilatifard [40] recently discussed the 'cause or cog' role of histone modifications in gene transcription, and proposed that histone modification patterns are actually the result of a series of dynamic processes coupled with transcription, including transcription factor binding, RNA polymerase elongation, nucleosome remodeling, and targeting of non-coding RNAs.

It has been shown that chromatin features possess a certain level of redundancy and that certain chromatin features may work in a combinatorial fashion. One way to study the effect of combinatorial chromatin features is to introduce interaction terms in the linear regression model, which is computationally expensive for a model with more than ten terms and has been shown to provide little contribution in improving the expression prediction accuracy [11]. Instead, we grouped chromatin features into different categories according to their known function in transcriptional regulation and performed regression on each category. This is less computationally expensive and the results are straightforward to understand. For example, grouping H3K4me2, H3K4me3, H2A.Z, and H3K27ac together allows us to determine how predictive promoter marks are for gene expression. However, the details of how these multiple chromatin features work together to reflect the gene expression levels need further exploration.

The model can be further improved in several ways. While the model can well predict gene expression using the current available set of chromatin features, we could retrain the model by incorporating newly discovered marks (such as histone lysine crotonylation [41]) and therefore study the importance of new effectors in regulating gene expression levels. Although our model shows good results for genes with single transcripts (Figure S11 in Additional file 2), multiple transcripts from the same gene may be subject to differential chromatin-based regulation. It is interesting and challenging to interpret chromatin-based regulation for multiple transcripts with shared TSSs. In this study, we chose the transcript with the highest expression level as the representative if a gene has multiple transcripts, which could hamper our ability in uncovering the effectors of repressed genes or transcripts (for example, a repressive mark such as H3K37me3). Also, if a gene has zero (or low) expression, we cannot tell whether it is unexpressed or suppressed. Unlike active marks (where a higher signal level indicates a higher expression level), repressive marks cannot lead to a negative expression level. These limitations could potentially underestimate the relative importance of repressive marks, which underscores a need for future work on refining the models for repressed genes. We have shown the general application of models across different cell types. As an extension of this analysis, further work could include building models to relate differential gene expression with differential histone modification profiles, and evaluate the relative contributions of these modifications to differential expression between cell types (for example, in differentiated versus H1-hESC cells). Due to the requirements of our binning method, we only included transcripts longer than 4,100 bp in this study. Also, current analysis only includes experiments for RNA molecules longer than 200 nucleotides. This leaves room for improvement in understanding how chromatin features help regulate other genes (especially long or short non-coding RNA genes). With regular improvements in gene annotation and expression quantification techniques, it is promising that we will understand the regulation of gene expression more accurately in the future.

## Conclusions

In this study, we have developed a novel two-step model to study the quantitative relationship between chromatin features and gene expression. We recapitulated previous findings that histone modifications are predictive of gene expression, and HCP and LCP genes are best predicted by different histone marks. Our model is generally applicable across multiple cell lines, and has led to several new insights, including: 1) histone modifications such as H3K9ac and H3K4me3 are more important for identifying genes that are 'on' or 'off,' while histone modifications such as H3K79me2 and H3K36me3 are more important for regression of expressed genes; 2) expression levels measured by all three techniques (CAGE, RNA-PET, and RNA-Seq) are well-predicted by the model (median *r *ranges from 0.79 to 0.88), and, on average, expression measured with CAGE is better predicted by the model than expression measured with RNA-PET or RNA-Seq; 3) promoter marks (for example, H3K4me2, H3K4me3, H2A.Z, H3K9ac, and H3K27ac) are the most predictive for CAGE-based measurement of transcription initiation, while structural marks like H3K79me2 and H3K36me3 are more predictive for RNA-Seq expression data (which can measure the transcription elongation); 4) PolyA+ RNA is overall better predicted by chromatin features than PolyA- RNA; and 5) for expression levels measured with RNA-Seq in different cellular compartments, RNA from major functioning compartments (for example, cytosolic PolyA+ RNA and nuclear PolyA- RNA) is better predicted by the model than RNA from other less functioning compartments (for example, nuclear PolyA+ RNA and cytosolic PolyA- RNA).

## Materials and methods

### The two-step prediction model

We used a two-step model to predict the expression levels of GENCODE genes: 1) we constructed a random forests classification model to predict whether a promoter was expressed or not; and 2) we constructed a regression model (for example, linear regression, MARS, or random forests) to predict the expression level of a promoter. The two models were combined by setting the predicted values ŷ_i _= C(X_i_)*R(X_i_), where C(X_i_) is the results from the classification model (C(X_i_) = 1 if promoter X_i _is predicted to be expressed, and 0 otherwise), and R(X_i_) is the predicted value for promoter X_i _by the regression model.

The performance of the classification model, the regression model, and the combined two-step model were evaluated based on ten-fold cross-validation. Each dataset was divided into a training set (a third of genes) and a testing set (two-thirds of genes). We trained a model using the training set and then applied it to the testing set to make predictions. We used AUC to represent the accuracy of the classification model, which measured the AUC (sensitivity versus 1 - specificity of a classification model). For the regression model, the predictive accuracy was measured by the PCC between the predicted value and the experimental value (*r*), and RMSE:

$$RMSE=\sqrt{\sum_{i}{\left(y_{i}-{\hat{y}}_{i}\right)}^{2}/n}$$

### Input datasets and gene annotation

All datasets used in this study are from the ENCODE project [13]. Genome-wide locations of eleven histone modifications (H3K4me1, H3K4me2, H3K4me3, H3K27me3, H3K36me3, H3K79me2, H3K9me1, H3K9me3, H4K20me1, H3K9ac, and H3K27ac) and one histone variant (H2A.Z) were generated by the Broad/MGH ENCODE group using ChIP-Seq [42], and are available from the Gene Expression Omnibus (GEO; accession number GSE29611). DNase I hypersensitivity was measured genome-wide using the Digital DNaseI methodology [43], and can be accessed via GEO accession number GSE32970. Uniformly processed genome-wide signal tracks for these signals were downloaded in bigwig format from the ENCODE project website [13].

GENCODE TSSs are defined as the most 5' position of GENCODE transcripts that show no evidence of an incomplete coding sequence (CDS) 5' end (for example, CDS start not found; tag not present). Each GENCODE TSS can be shared by multiple GENCODE transcripts. From the 153,993 GENCODE v7 transcripts that fulfill the above criteria, we derived 137,958 GENCODE v7 TSSs, which we then quantified using three different technologies: CAGE, RNA-PET and RNA-Seq. Since CAGE captures the 5' ends of the transcripts, the CAGE expression of a given TSS is defined as the sum of the CAGE tags whose 5' end falls within the 101 bp window centered on the TSS. In order to compare TSS expression from different CAGE experiments, this expression is further normalized by the total number of mapped CAGE tags in the experiment and multiplied by 1 million (number of reads per million mapped reads (RPM) value). RNA-PET provides both the 5' and the 3' ends of transcripts, and the RNA-PET expression of a given TSS is defined as the sum of the RNA-PET 5' tags whose 5' ends fall within the 101 bp window centered on the TSS. Again this expression is normalized by the total number of mapped RNA-PET 5' tags in the experiment. For RNA-Seq experiments, we used GENCODE v7 transcript expression as measured by RPKM (reads per kilobase per million mapped reads; computed using the flux capacitor [44]) to measure GENCODE v7 TSS expression. If a TSS is shared by transcripts t_1_,... t_n_, its expression in an RNA-Seq experiment will be defined as the sum of the RPKM of transcripts t_1_,... t_n _in this same experiment (already normalized). This procedure assigns each RNA-Seq read (or each part of the read) to one transcript only, thus not counting it multiple times because the flux capacitor is a deconvolution tool. The raw data from expression profiling can be downloaded from the GEO (accession numbers GSE26284 (RNA-Seq), GSE34448 (CAGE), and GSE33600 (RNA-PET)).

As described previously [28], normalized CpG content for each transcript was calculated for the [-1,500 bp, +1,500 bp] region flanking the TSS. Promoters with normalized CpG content >0.4 are defined as HCP, and those with normalized CpG content ≤0.4 as LCP.

### Dealing with multiple replicates and genes with multiple transcripts

To reduce the possibility of bias from a single measurement, the ENCODE Consortium performed multiple biological replicates for most experiments. To reduce redundancy, we merged multiple replicates of the same experiment by taking the mean expression level of each gene from the replicates.

For genes with multiple transcripts, it is difficult to decipher which transcript is correlated with the signal of chromatin features. This may lead to bias, particularly in cases where the 'on' and 'off' transcripts have very close TSSs but different expression levels. To avoid this bias, we selected the transcript with the strongest expression level as the representative transcript for each gene.

### Defining the 'bestbin' of chromatin feature density

For each transcript longer than 4,100 bp, we extended the transcript by 2,000 bp on each side and divided it into 81 bins (40 bins for the [-2k, +2k] region flanking the TSS, one bin for the rest of the gene body, and 40 bins for the [-2k, +2k] region around the TTS). We calculated the mean density of chromatin features in each bin by using the bigWigSummary command-line utility [45]. We defined the 'bestbin' for each chromatin feature as the bin with the highest absolute correlation coefficient with gene expression levels. For Tx-based expression data, we searched for the 'bestbin' among all 81 bins. For TSS-based expression data such as CAGE, we could not tell which transcript the CAGE tags were from if multiple transcripts shared the same TSS, so we used 41 bins for each unique TSS (that is, the first 40 bins plus one bin of the gene body from the above 81 bins) to ensure full coverage of the relevant chromatin feature signals.

### Data transformation and pseudocount optimization

Because log2 transformation was applied to the signal of chromatin features X*_ij _*for each gene *i *and chromatin feature *j*, a small pseudocount a*_j _*was added to the values of each chromatin feature to avoid the log_2_(0) issue. We used one-third of the genes in each dataset to optimize the pseudocount, and applied the optimized pseudocount to the remaining two-thirds of the genes. For each bin of chromatin feature *j*, we searched for the optimized pseudocount a*_j_*ranging from 0 to 20% of the maximal value of X*_ij _*in that bin. The optimized pseudocount a*_j _*was determined by a maximal correlation between log_2_(X*_ij _*+ a*_j_*) and logarithm of measured expression values for one-third of the genes in each dataset.

As an alternative to log transform and using pseudocounts, we also converted data to 'normal scores' using rankit transformaton, which samples the same number of values from an equivalent normal distribution, followed by re-ordering of the data. We implemented the rankit transformation in R as:

x= qnorm((rank(x) - 0.375)/(sum(!is.na(x)) + 0.25))

### Variable importance

For the linear regression model, we used the R^2 ^decomposition according to Verena and Korbinian [46] implemented in the calc.relimp function in the {relaimpo} R package. For MARS, we used the nsubsets criterion implemented in the evimp function in the {earth} R package [47], which counts the number of model subsets that include the variable of interest. Variables that are included in a greater number of subsets are considered more important. For random forests, we used the decreased Gini index as criteria of variable selection [48], which was implemented in the importance function of the {randomForest} R package.

## Abbreviations

AUC: area under the receiver operating characteristic curve; bp: base pair; CAGE: cap analysis of gene expression; GEO: Gene Expression Omnibus; HCP: high CpG content promoter; LCP: Low CpG content promoter; MARS: multivariate adaptive regression splines; PCC: Pearson's correlation coefficient; RMSE: root-mean-square error; RNA-PET: RNA paired-end tag; ROC: receiver operating characteristic; RPKM: reads per kilobase per million mapped reads; TSS: transcription start site; Tx: transcript.

## Competing interests

The authors declare that they have no competing interests.

## Authors' contributions

XD carried out the studies and drafted the manuscript. MCG helped formalize the manuscript. AK, JB, CC, and MG participated in the modeling. TG, SD and RG generated the expression data. EB participated in the modeling and design of the study. ZW conceived of the study, and participated in its design and coordination and helped to write the manuscript. All authors read and approved the final manuscript.

## Supplementary Material

Additional file 1 **Supplementary tables**. Table S1: bestbin and pseudocount results for each mark. Table S2: results of all predictions, including the correlation coefficient, *P*-value for the correlation, the individual correlation, and relative importance of each chromatin feature. Table S3: list of experiments used in the analysis. Click here for file

Additional data file 2 **Supplementary figures**. Figure S1: model diagnosis. **(A) **ROC curve for random forests classifier in predicting the 'on' and 'off' expression status for the CAGE PolyA+ cytosolic RNA from K562 cells. The AUC (area under the curve) is 0.95 and error rate is 9.56%. **(B) **Residual plot for the fitted values. The red line is the mean of residuals, which should be centered around 0 for a model without systematic bias. The sharp border at the bottom of the scatter plot is due to the limited resolution of measured expression (for example, not enough data points between 0 and first non-zero value). **(C) **Q-Q plot of standardized residuals, which shows that standardized residuals are normally distributed. **(D) **Scatter plot of predicted expression and measured expression using the 'rankit' transformation (which samples from an equivalent normal distribution that respects the rank order of the expression data; see Materials and methods). PCC *r *= 0.86 for overall prediction (*P*-value <2.2 × 10^-16^), AUC for classification is 0.94 and PCC *r *for regression is 0.72. Figure S2: comparison of the performance of three regression models. Figure S3: model stability. Each bar is a set of randomly sampled genes (10%, 20%,... 100% of all genes). The blue line represents the PCC *r *for each set. The black line with filled circles is the percentage of high-CpG promoter (HCPs) genes and the open circle black line is the percentage of low-CpG promoter (LCPs) genes in each set. The model performance is stable regardless of sample size. Figure S4: comparison of performance between HCP and LCP genes. **(A,B) **The performance of different chromatin feature categories for predicting HCP genes versus LCP genes (A) and highly expressed versus lowly expressed genes (B). It shows the results of the top X% of genes (X =10, 20, 30,... 100) in decreasing order of expression for CAGE PolyA+ cytosolic RNA from K562 cells. Figure S5: heatmap of correlation between replicates of expression experiments. Among the total of 98 experiments, 55 experiments have two biological replicates (replicates 1 and 2). The heatmap indicates that two replicates from the same technique, RNA type, cell line, and compartment are generally highly correlated. Code for RNA type: t, total RNA; +, PolyA+; -, PolyA-. Code for cell lines: K, K562; G, GM12878; 1, H1-hESC; H, HepG2; E, HeLa-S3; N, NHEK; U, HUVEC. Code for cell compartment: W, whole cell; C, cytosol; N, nucleus; h, chromatin; u, nucleolus; l, nucleoplasm. Figure S6: heatmap of correlation between CAGE and RNA-Seq experiments for single-transcript genes. Each row (or column) depicts a PolyA+ RNA expression experiment from one of the cellular compartments (cytosol, nucleus, and whole cell) and one of seven cell lines (H1-hESC, HeLA-S3, GM12878, HepG2, K562, NHEK, and HUVEC) from CAGE or RNA-Seq. It shows that CAGE and RNA-Seq expression from the same cell lines are well-correlated (black-frame boxes), even though the correlation is weaker than experiments using same quantification method (the red blocks along the diagonal). There are a total of 31,484 genes with single transcripts. Figure S7: model performance using DNase I hypersensitivity only and promoter marks only. Each bar is the correlation coefficient of predicting expression using only either DNase I hypersensitivity or promoter marks (that is, H3K4me2, H3K4me3, H2A.Z, H3K9ac, and H3K27ac). It shows that promoter marks are more predictive than DNase I hypersensitivity (paired Wilcoxon test *P*-value = 4 × 10^-15^). Figure S8: heatmap of correlations between PolyA+ RNA-Seq and PolyA- RNA-Seq. Figure S9: stability of the 'bestbin' selection. Each panel is a histogram of the 'bestbin' index for a chromatin mark. Since the 'bestbin' is calculated based on a randomly selected one-third of the total dataset (D1 in Figure 1) for each experiment, the most stable 'bestbin' will be shown as a sharp peak on the histogram. Figure S10: improvement by pseudocount optimization. Correlation coefficient of histone modification (H3K79me2) density with expression level is calculated at each bin, using a fixed pseudocount of 0.001 or an optimized pseudocount (see Materials and methods). The pseudocount optimization (black line) consistently performs better than the fixed pseudocount (gray line). The blue line indicates the average H3K79me2 level. Figure S11: prediction using single-transcript genes. PCCs (*r*) of all 78 RNA expression experiments using only the single-transcript gene subset. Comparing Figures S10 and 2c, we can see that there is no significant change in model performance or most important variables when including genes with multiple transcripts. Click here for file
