# Supplementary figures and images for: Modeling gene expression using chromatin features in various cellular contexts
Source: Genome Biol. 2012 Sep 5;13(9):R53. doi: 10.1186/gb-2012-13-9-r53 (PMC3491397; doi:10.1186/gb-2012-13-9-r53)

A

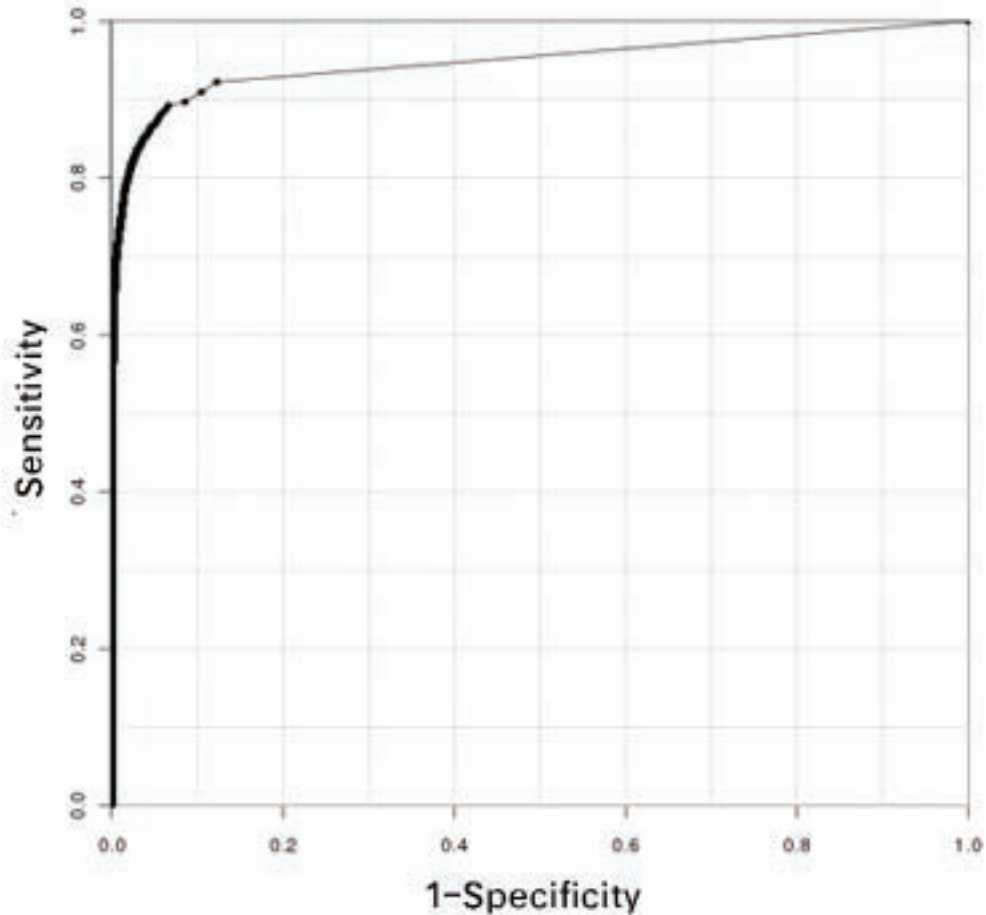

B

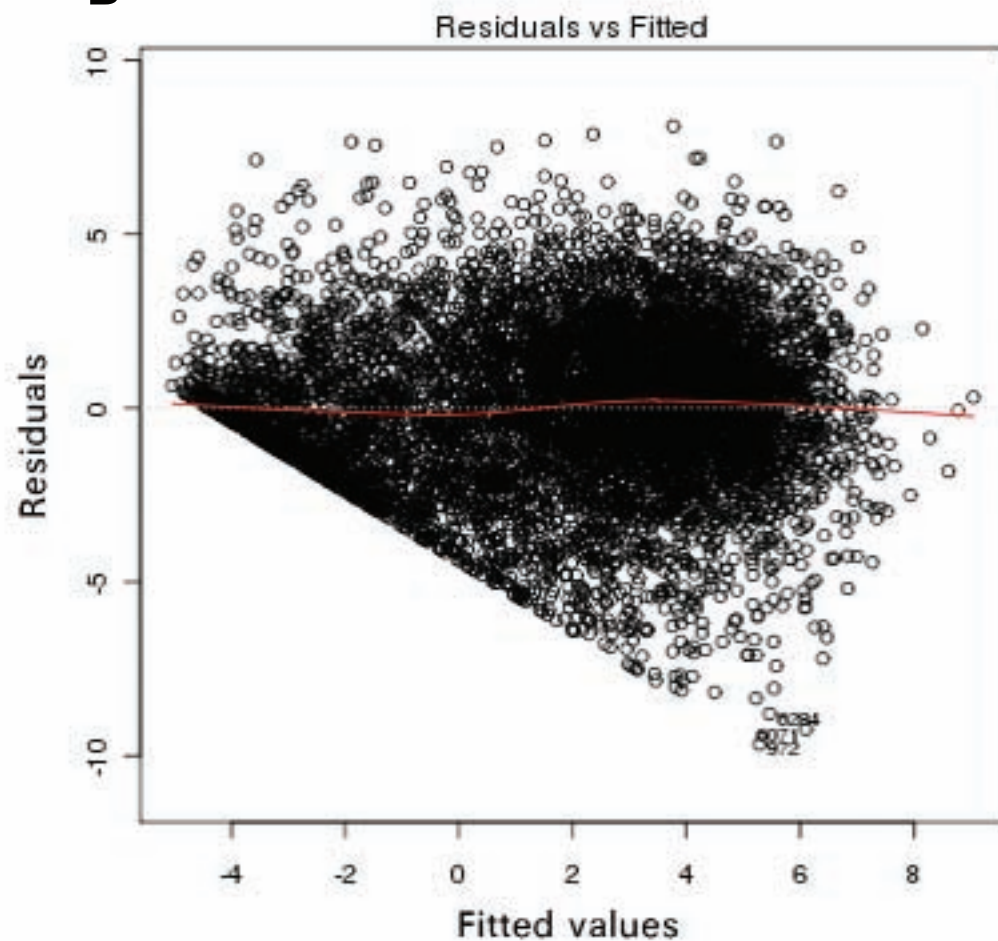

C

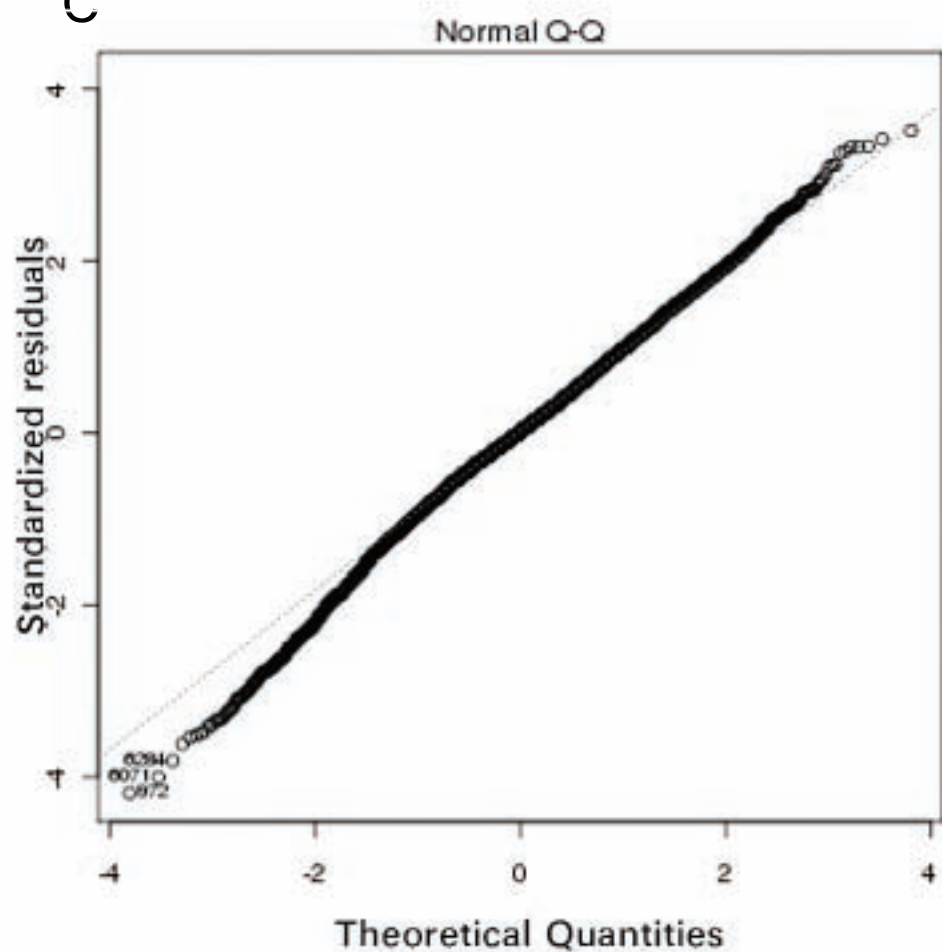

D

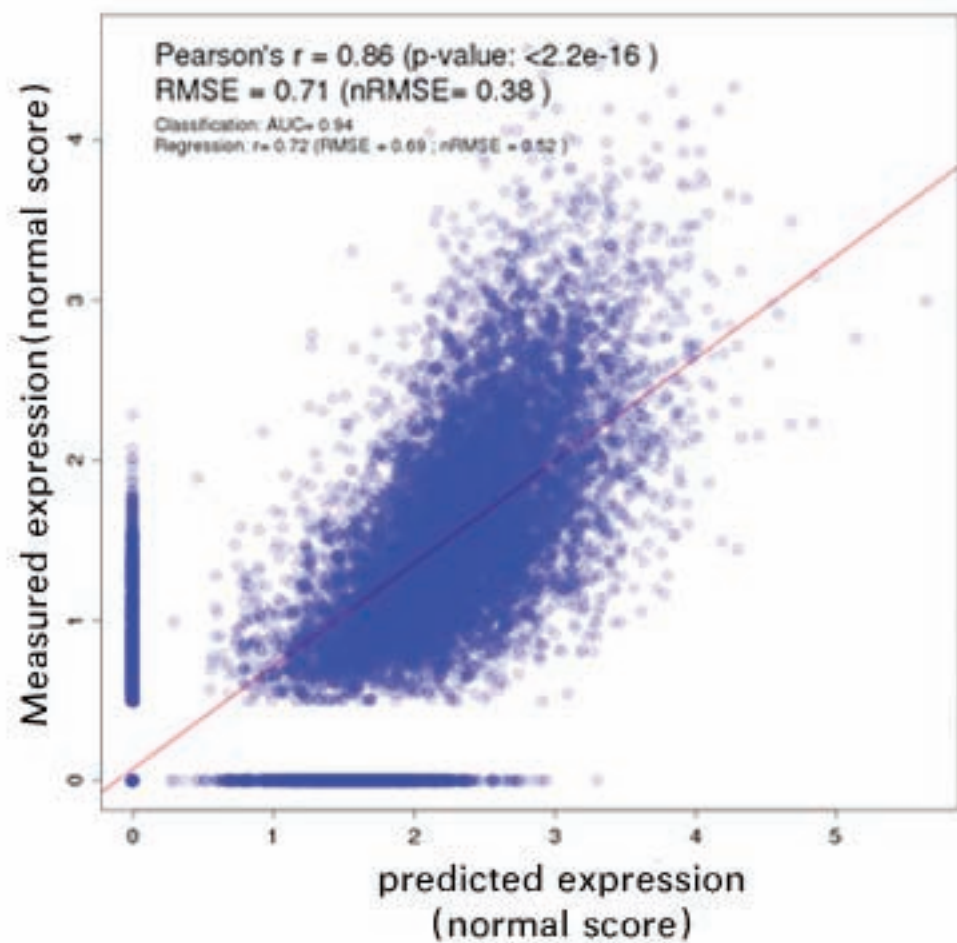

## Comparison of modelling methods

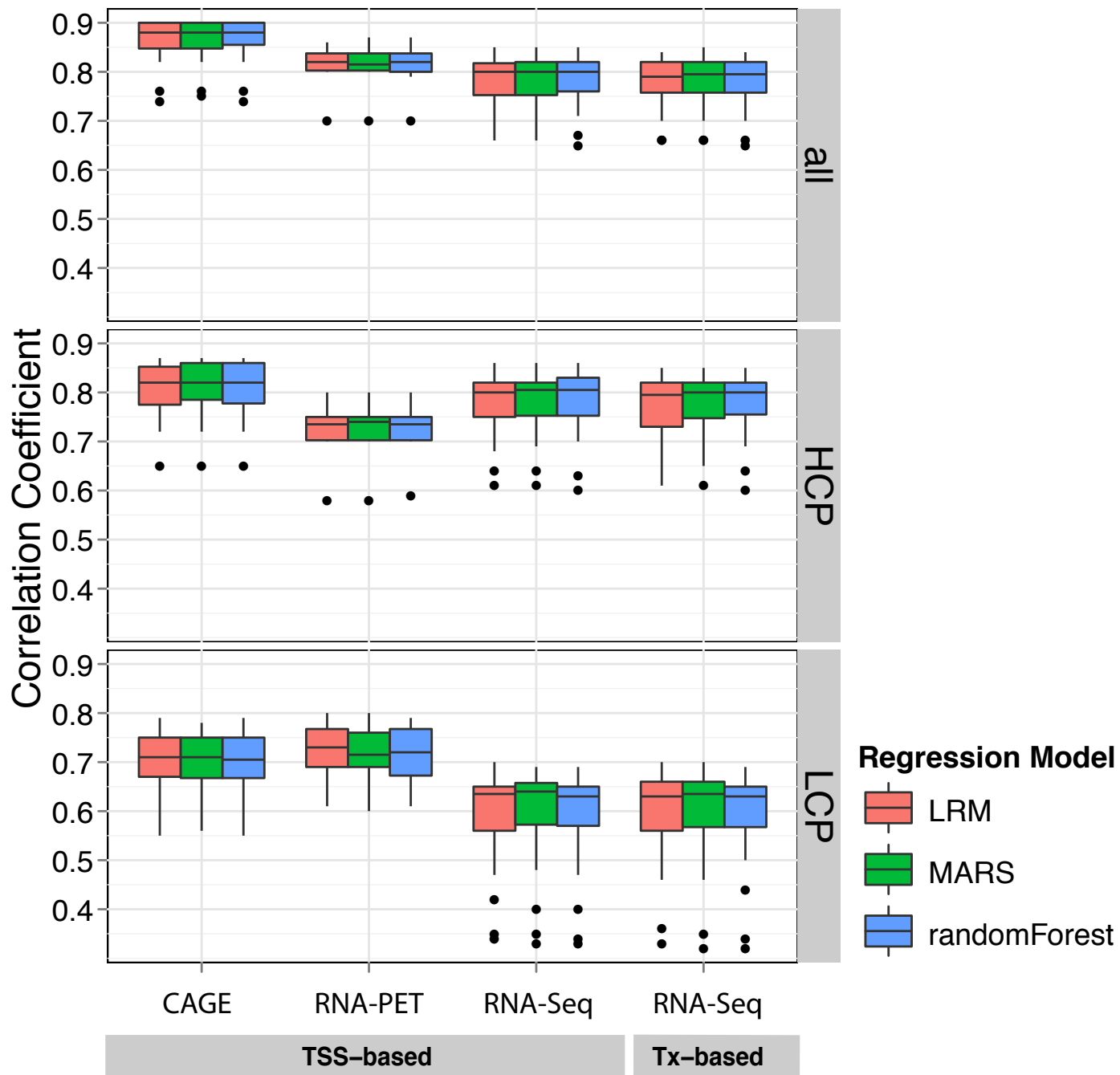

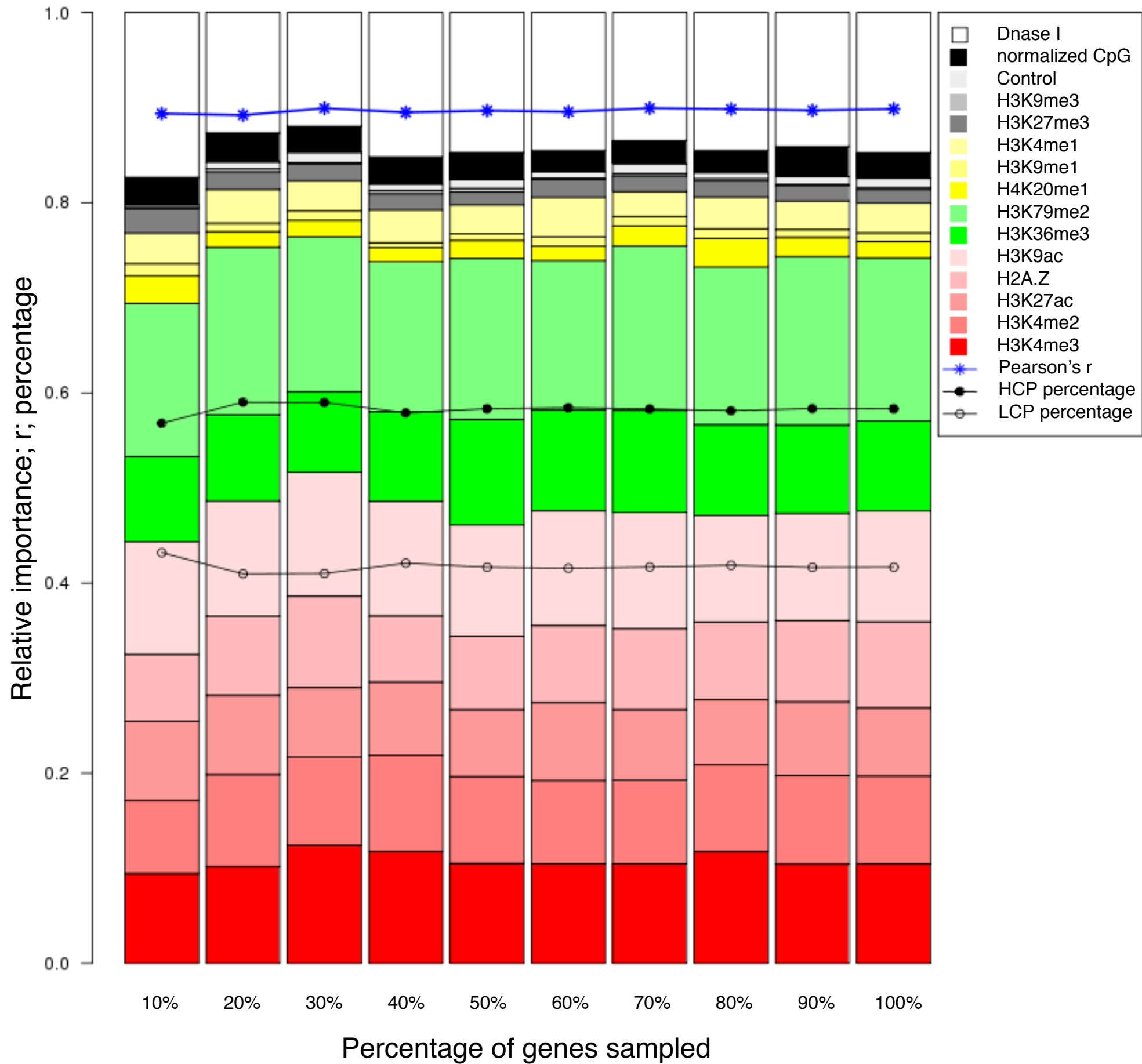

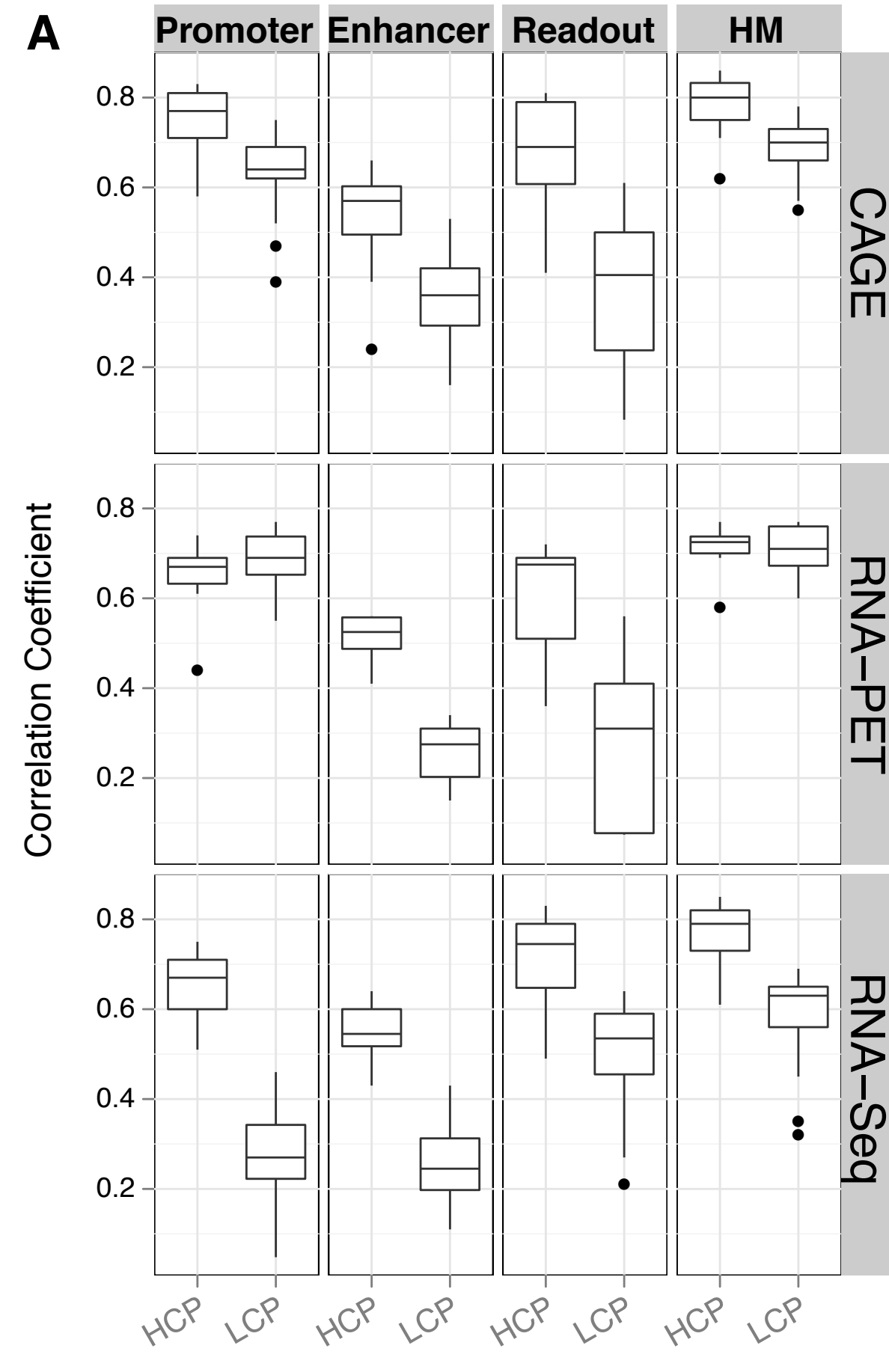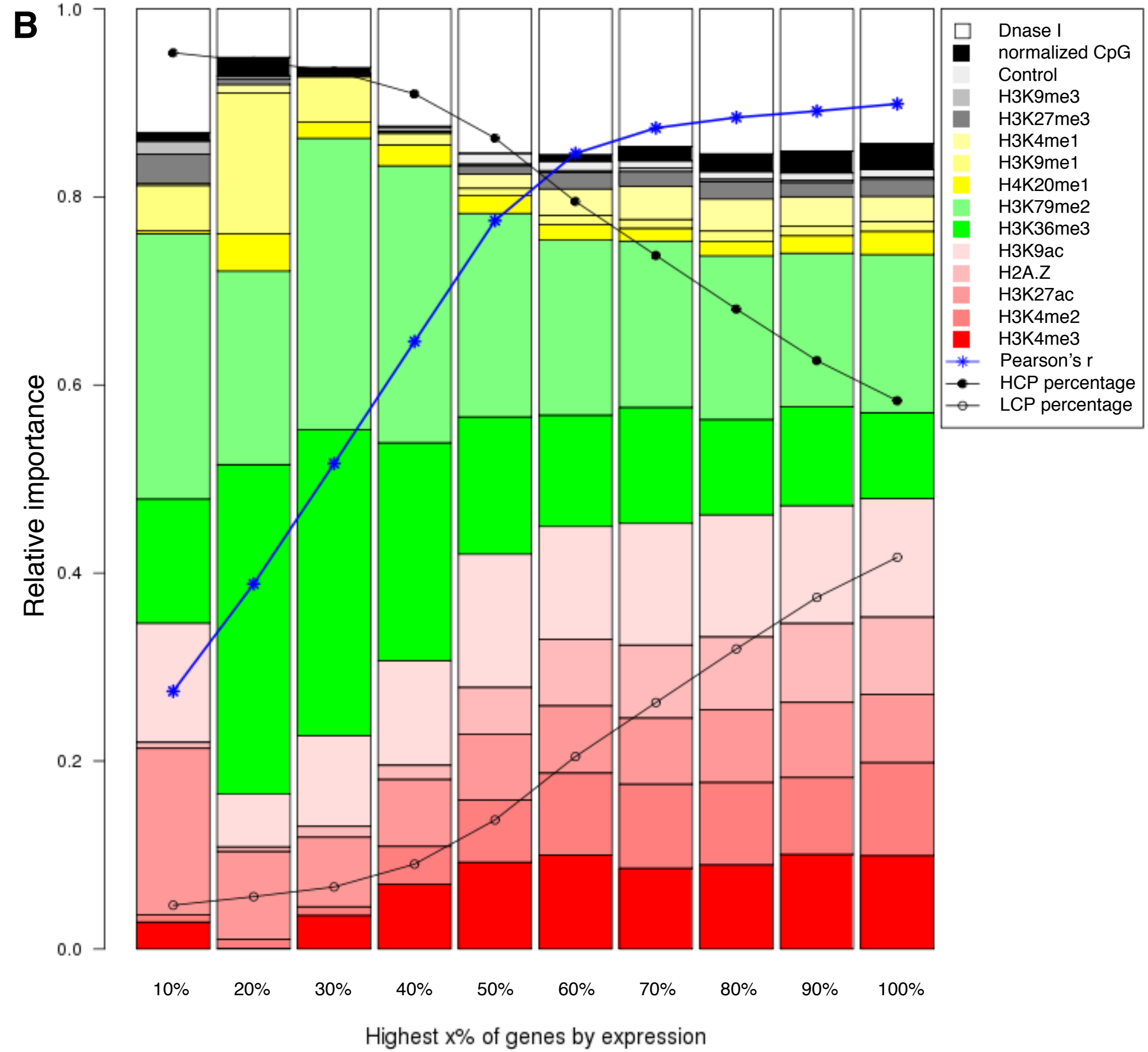

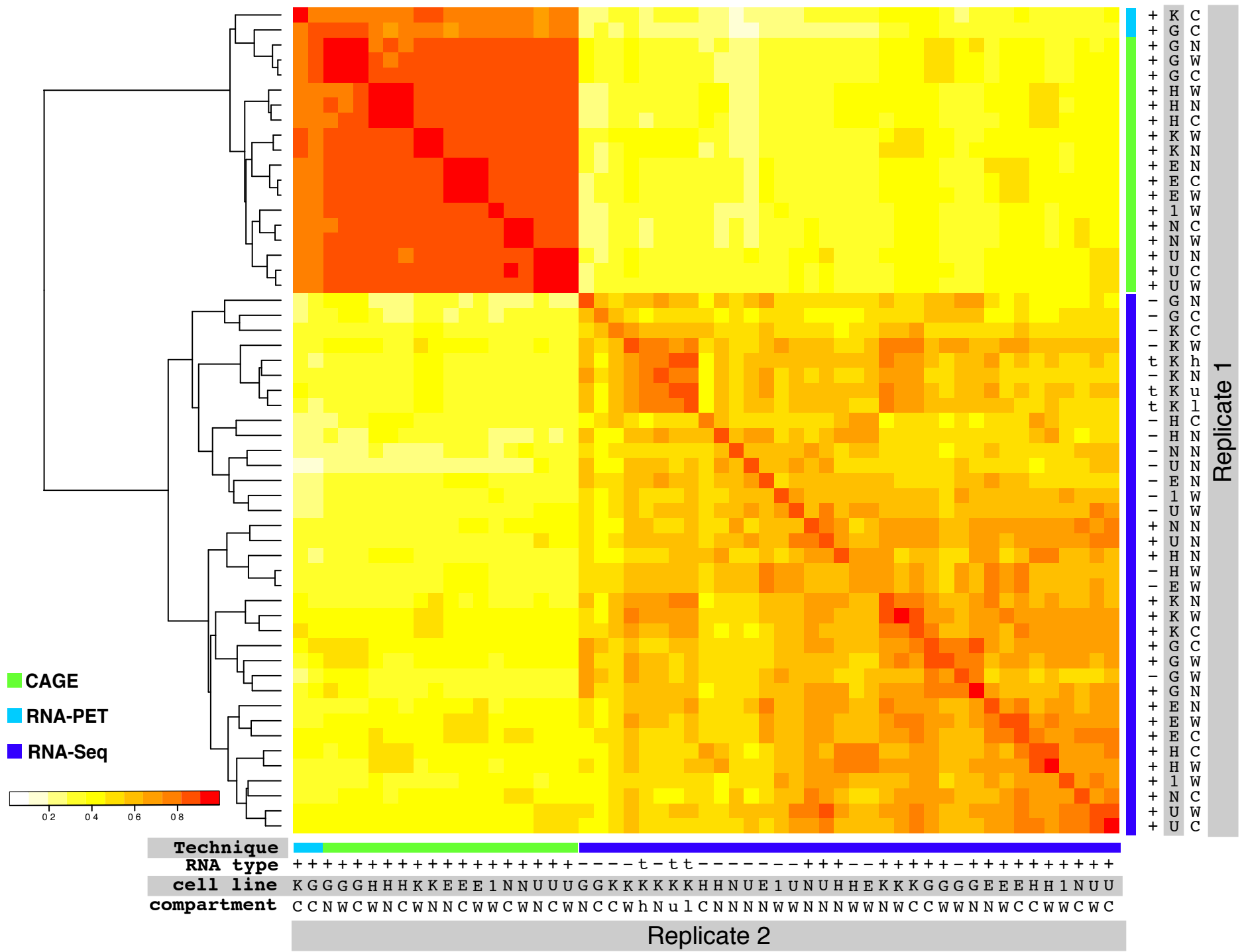

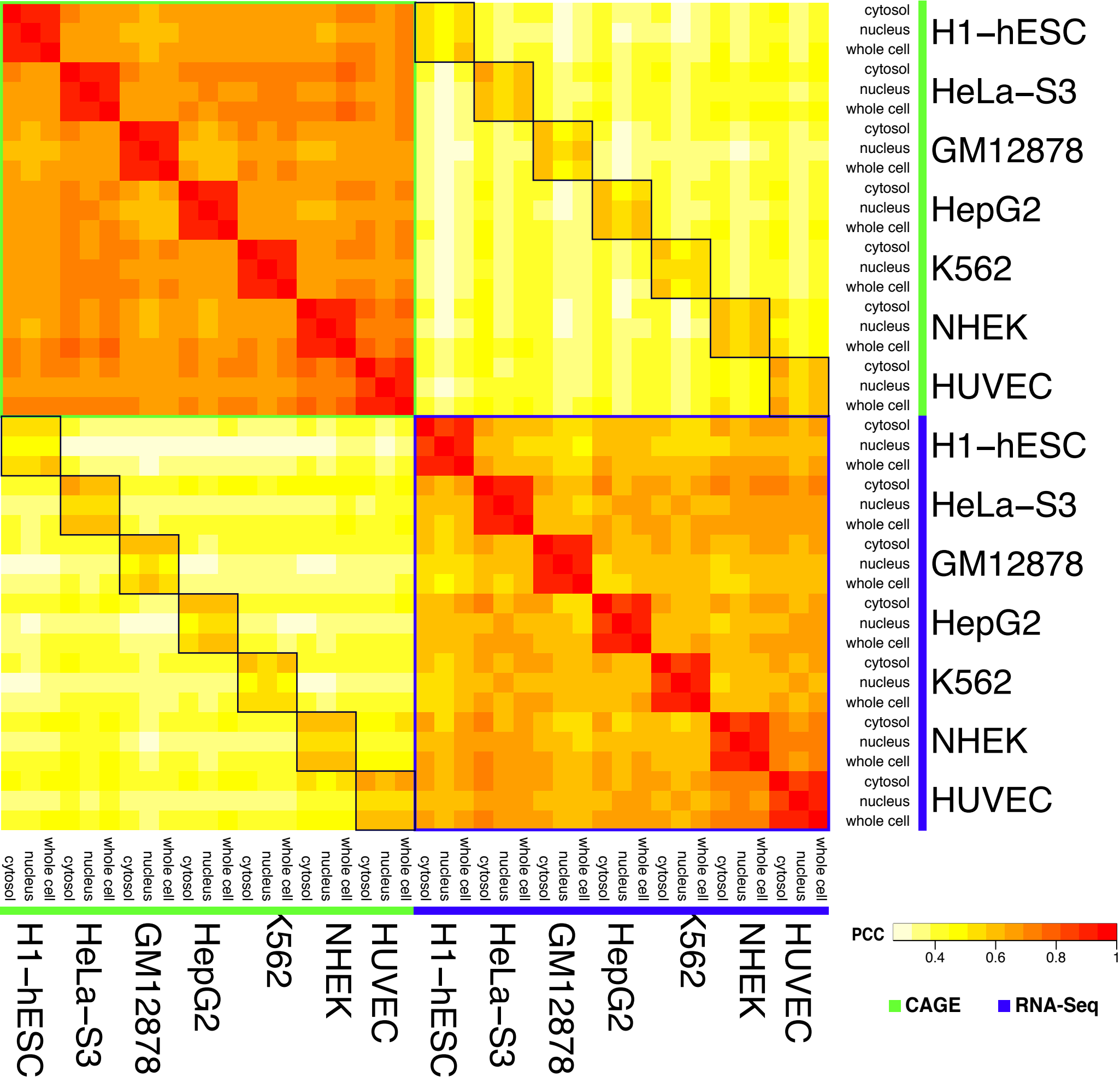



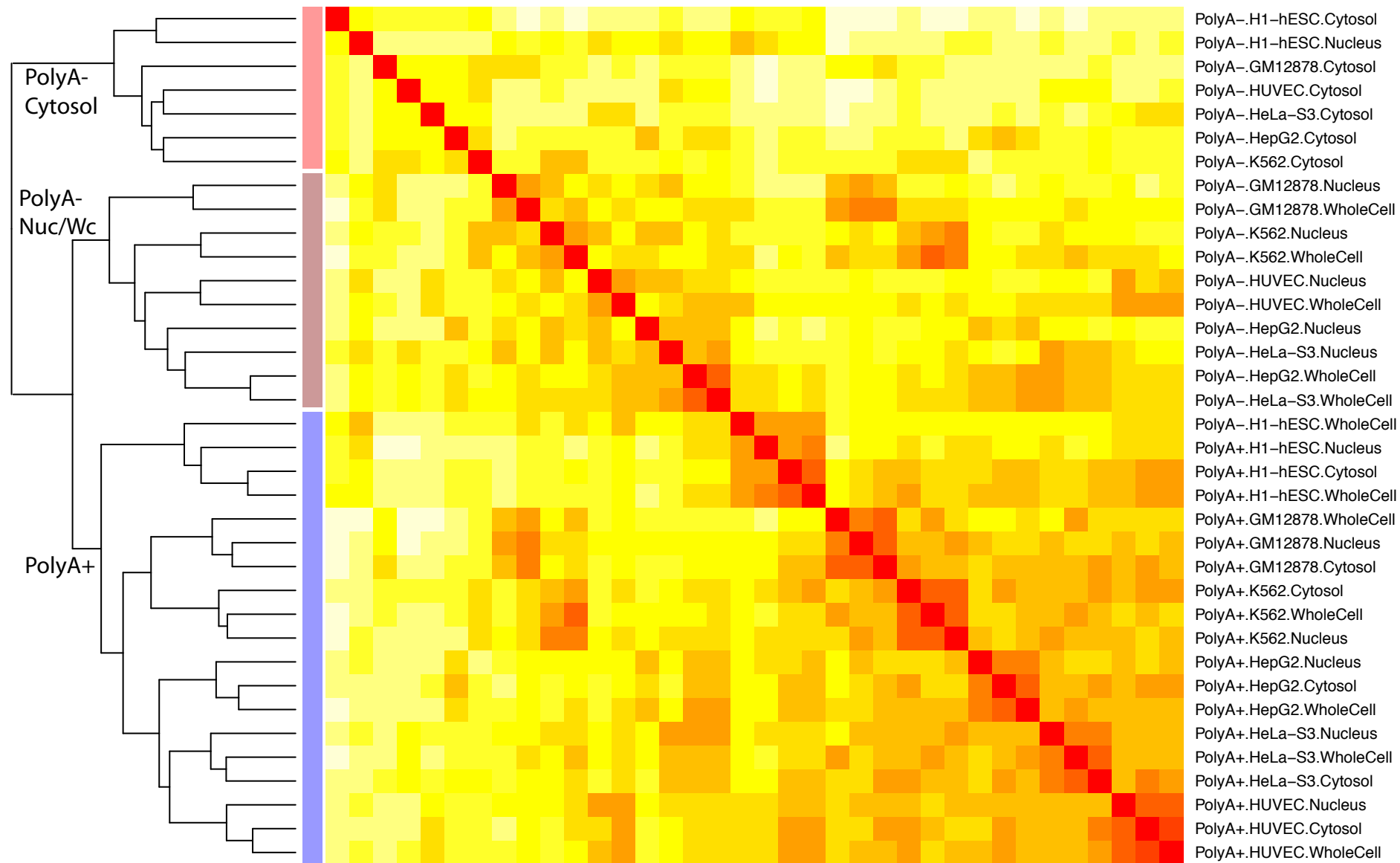

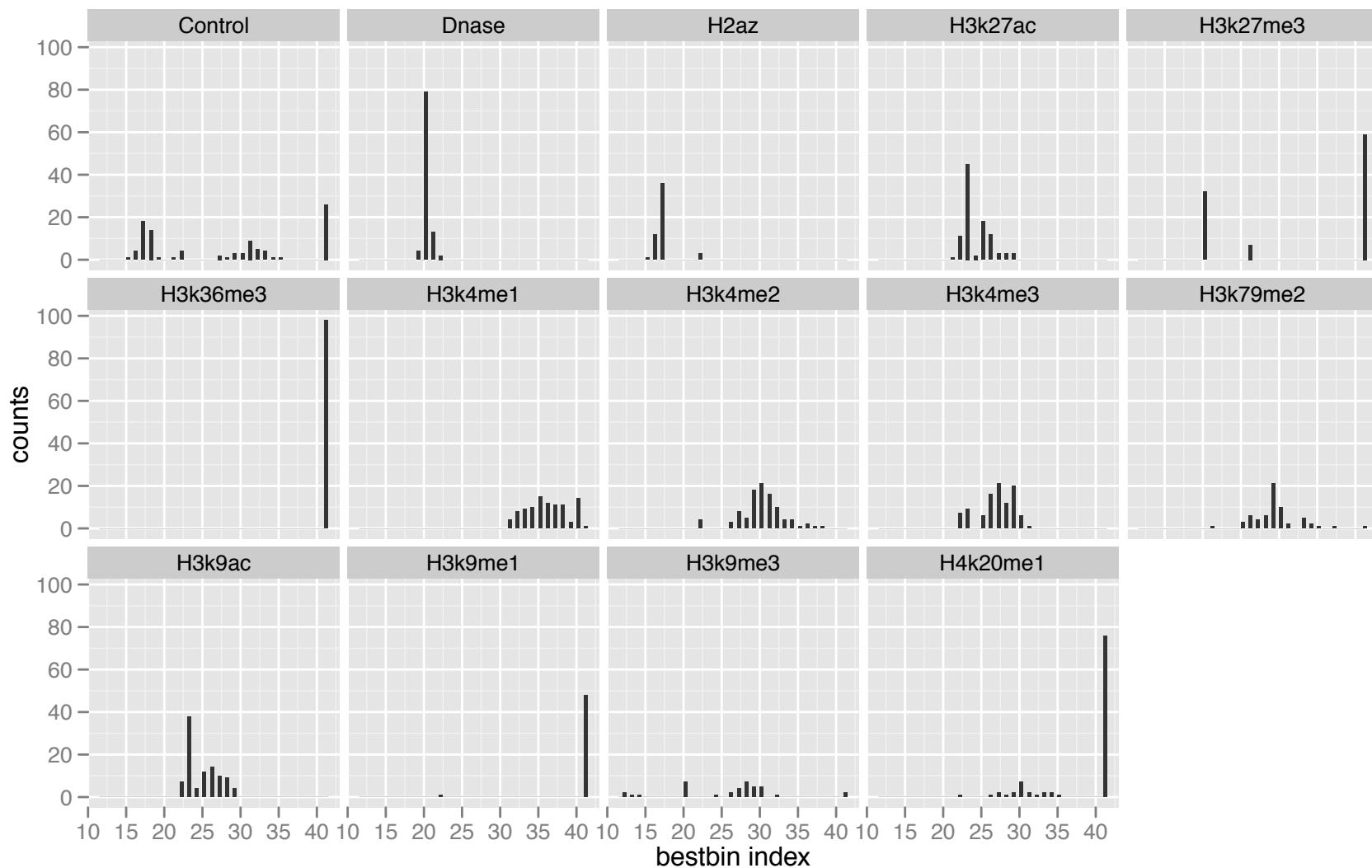

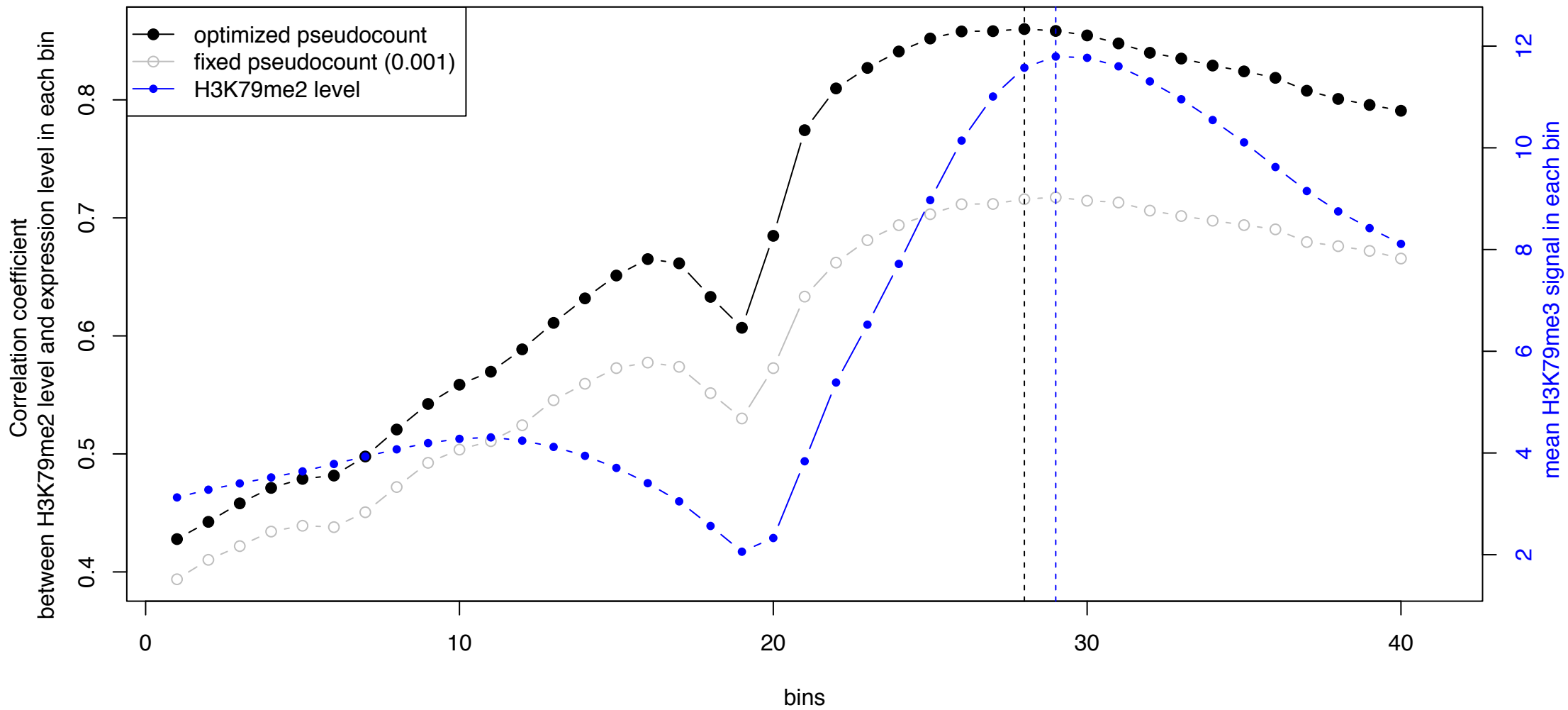

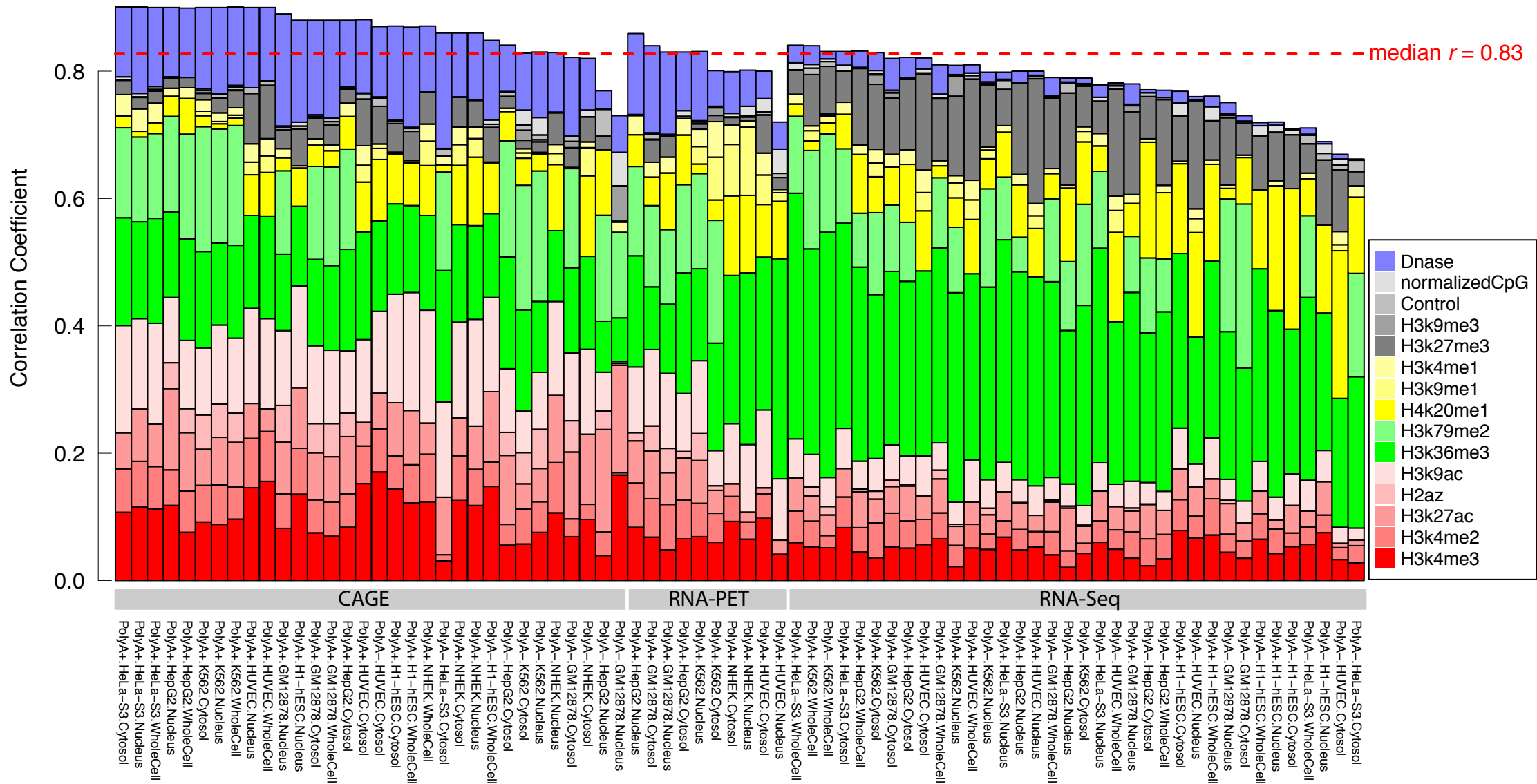

Supplement: Additional data file 2 — Supplementary figures. Figure S1: model diagnosis. (A) ROC curve for random forests classifier in predicting the 'on' and 'off' expression status for the CAGE PolyA+ cytosolic RNA from K562 cells. The AUC (area under the curve) is 0.95 and error rate is 9.56%. (B) Residual plot for the fitted values. The red line is the mean of residuals, which should be centered around 0 for a model without systematic bias. The sharp border at the bottom of the scatter plot is due to the limited resolution of measured expression (for example, not enough data points between 0 and first non-zero value). (C) Q-Q plot of standardized residuals, which shows that standardized residuals are normally distributed. (D) Scatter plot of predicted expression and measured expression using the 'rankit' transformation (which samples from an equivalent normal distribution that respects the rank order of the expression data; see Materials and methods). PCC r = 0.86 for overall prediction (P-value <2.2 × 10^-16^), AUC for classification is 0.94 and PCC r for regression is 0.72. Figure S2: comparison of the performance of three regression models. Figure S3: model stability. Each bar is a set of randomly sampled genes (10%, 20%,... 100% of all genes). The blue line represents the PCC r for each set. The black line with filled circles is the percentage of high-CpG promoter (HCPs) genes and the open circle black line is the percentage of low-CpG promoter (LCPs) genes in each set. The model performance is stable regardless of sample size. Figure S4: comparison of performance between HCP and LCP genes. (A,B) The performance of different chromatin feature categories for predicting HCP genes versus LCP genes (A) and highly expressed versus lowly expressed genes (B). It shows the results of the top X% of genes (X =10, 20, 30,... 100) in decreasing order of expression for CAGE PolyA+ cytosolic RNA from K562 cells. Figure S5: heatmap of correlation between replicates of expression experiments. Among the total of [file gb-2012-13-9-r53-S2.pdf]
